# Endometriosis as an immune-mediated disease: pathogenetic mechanisms and therapeutic strategies

**DOI:** 10.3389/fimmu.2025.1727183

**Published:** 2025-12-18

**Authors:** Sofia Shifon, Tamara Tyrinova, Tatyana Veretelnikova, Natalia Pasman, Elena Chernykh

**Affiliations:** 1 Institute of Medicine and Medical Technologies, Novosibirsk National Research State University, Novosibirsk, Russia; 2 Laboratory of Cellular and Molecular Mechanisms of Immunopathology, Research Institute of Fundamental and Clinical Immunology, Novosibirsk, Russia; 3 Laboratory of Cellular Immunotherapy, Research Institute of Fundamental and Clinical Immunology, Novosibirsk, Russia; 4 Department of Immunology, Faculty of Medicine and Psychology, Novosibirsk National Research State University, Novosibirsk, Russia; 5 Gynecology Department “Professor Pasman Clinic”, Novosibirsk, Russia; 6 Department of Obstetrics and Gynecology, Faculty of Medicine and Psychology, Novosibirsk National Research State University, Novosibirsk, Russia

**Keywords:** autoimmunity, complement system, cytokines, endometriosis, immunopathogenesis, immunotherapy, inflammation, macrophages

## Abstract

Endometriosis, which affects approximately 10% of women of reproductive age, is a complex inflammatory disease with significant immune system disturbances caused by an inadequate immune response to retrograde menstruation and leading to the establishment of immune evasion mechanisms by ectopic tissue. This review provides an analysis of the immunopathogenetic mechanisms of endometriosis based on 198 high-quality publications selected from 1,209 potentially relevant articles in the PubMed, Scopus, Web of Science, and Google Scholar databases for the period 1927–2025. The study revealed that endometriosis is associated with profound alterations in both innate and adaptive immunity. Key pathogenetic mechanisms include macrophage dysfunction with a shift to the M2 phenotype, reduced cytotoxic activity of NK cells, complement system activation with proinflammatory and proangiogenic effects, a predominant Th2 response with an increase in Treg cells, and B-lymphocyte activation with autoantibody production. The cytokine profile is characterized by a concurrent increase in both pro-inflammatory mediators (IL-1β, IL-6, TNF-α) and immunosuppressive factors (IL-10, TGF-β). The complement system contributes to pathogenesis through C3a/C5a-mediated inflammation, angiogenesis promotion, and interactions with dysbiotic endometrial microbiota. Different forms of endometriosis have specific immunological features: ovarian endometriosis combines local immunosuppression with systemic inflammation, adenomyosis is characterized by pro-inflammatory changes with a Treg cell deficiency, and deep infiltrating endometriosis is distinguished by the activation of the IDO1/COX-2/MMP-9 signaling pathway and complement-mediated tissue destruction. Understanding the specifics of immunopathogenesis opens new avenues for developing targeted immunotherapy, which may include modulating immune cell functions, using cytokine inhibitors, blocking immune checkpoints, and employing nanotechnological approaches.

## 1 Introduction

Endometriosis is one of the most prevalent gynecological diseases, affecting up to 10% of women of reproductive age worldwide (1). Endometriosis encompasses two distinct forms: external endometriosis, characterized by ectopic lesions outside the uterus, and internal endometriosis (adenomyosis), in which endometrial tissue penetrates into the myometrium. External endometriosis manifests in various anatomical locations, including the peritoneum, ovaries (endometriomas), rectovaginal septum (deep infiltrating endometriosis), and extragenital sites—intestine, bladder, lungs, surgical scars (1) (from the existing list). The theory of retrograde menstruation, first described by Sampson in 1927, proposes that menstrual debris containing viable endometrial cells is refluxed through the fallopian tubes and implanted on pelvic structures (2). However, since retrograde menstruation is observed in 90% of women, while endometriosis develops in only 10%, additional factors must determine susceptibility to the disease (3). Alternative theories include coelomic metaplasia (transformation of peritoneal mesothelium into endometrium-like tissue), lymphovascular dissemination (explaining distant lesions), and the stem/progenitor cell theory (4). For adenomyosis, proposed mechanisms include invagination of the basal endometrium into the myometrium, the tissue injury and repair (TIAR) hypothesis, and *de novo* development from Müllerian remnants (5). Current understanding recognizes endometriosis as a multifactorial disease involving genetic predisposition, hormonal influences, environmental factors, and—critically—immune dysfunction, which is the focus of this review. Importantly, these pathogenetic mechanisms should not be viewed as mutually exclusive alternatives, but rather as complementary components of a unified multistep process. A synthetic hypothesis integrating the cumulative roles of various mechanisms suggests that retrograde menstruation provides the initial seeding of endometrial cells, while genetic susceptibility, hormonal milieu, and environmental exposures determine whether these cells survive. Crucially, immune dysfunction serves as the permissive factor that allows ectopic implants to evade clearance, establish vascularization, and progress to clinically significant disease. This integrative framework underscores the necessity of understanding how these mechanisms converge and synergize in the pathogenesis of endometriosis.

The pathology is characterized by the growth of tissue similar in morphological and functional properties to endometrium outside the uterine cavity. The principal clinical manifestations of endometriosis include chronic pelvic pain, dysmenorrhea, dyspareunia, and infertility (6). Despite significant research efforts, the etiology and pathogenesis of endometriosis remain incompletely understood.

In recent years, increasing attention has been devoted to the role of the immune system in the development and progression of endometriosis. Numerous studies, including domestic research, have demonstrated that women with endometriosis exhibit substantial disruptions of both innate and adaptive immunity (7). These alterations include dysfunction of macrophages, NK cells, T- and B-lymphocytes, as well as imbalances in the production of pro- and anti-inflammatory cytokines (8). Understanding the immunopathogenesis of endometriosis opens new perspectives for developing targeted (selective) therapy directed at correcting identified immune disruptions.

In endometriosis, immune system alterations manifest both at the local level (in ectopic lesions and peritoneal fluid) and as systemic changes in peripheral blood (8, 9). However, the relationship between local and systemic disruptions remains incompletely clear. In advanced stages of endometriosis, immune disruptions are more pronounced, whereas in early stages they may be less apparent (10). This complicates early diagnosis of the disease and the use of immune parameters as biomarkers for evaluating therapeutic efficacy. Identification of universal immunological biomarkers in endometriosis is also complicated by disease heterogeneity and potentially different immunopathogenetic mechanisms in different clinical forms (11, 12). Finally, result variability may be attributed to previous therapy, differences in inclusion/exclusion criteria, and differences in applied methods for immune status evaluation (flow cytometry, enzyme-linked immunosorbent assay [ELISA], polymerase chain reaction [PCR], and others).

Thus, further research into the immunopathogenesis of endometriosis should consider the local-systemic nature of the process, the degree of immune changes at different stages and in different clinical forms, the influence of previous therapy, methodological aspects, and the contribution of individual immune system components for a more complete understanding of the mechanisms of endometriosis development and progression. Therefore, it is crucial to evaluate the current approaches to understanding the immunopathogenesis of endometriosis.

The immunological aspects of endometriosis have been addressed in several recent reviews. Symons et al. (12) provided a comprehensive analysis of the immunopathophysiology of endometriosis, highlighting the role of defective immune surveillance in ectopic cell survival and discussing the potential for immunotherapy. Riccio et al. (8) focused on the aberrant immune response within the peritoneal microenvironment and the mechanisms that allow ectopic endometrial cells to evade apoptosis and immune surveillance.

Zhang et al. systematized data on the relationship between immunity, autoimmunity, and endometriosis, with a particular focus on autoantibodies and cytokines (13). Abramiuk et al. (14) summarized the role of various components of the immune system in the development of endometriosis, emphasizing cellular mechanisms. However, existing reviews either focus on specific aspects of immunopathogenesis or fail to account for the clinical and morphological heterogeneity of the disease. The present review is characterized by a comprehensive and structured approach: it sequentially analyzes impairments across all key immune cell populations of the innate and adaptive immunity and the role of soluble inflammatory mediators in forming the pathological microenvironment. This analysis allows for the identification of mechanisms underlying the bidirectional interaction between the immune system and endometriotic lesions. These insights are integrated through an examination of the immunological features of different clinical forms of endometriosis, followed by an analysis of prospects for immune therapy.

The objective of this review is to systematize and synthesize contemporary understanding of the immunopathogenesis of endometriosis, as well as to discuss potential immune-targeted therapeutic approaches for this disease.

## 2 Innate immunity in endometriosis

### 2.1 Neutrophils and their role in inflammation chronicity

Neutrophils are key cells of innate immunity and among the first to respond to tissue damage and inflammation. In endometriosis, increased neutrophil infiltration of peritoneal fluid and endometriotic lesions is observed. This may be associated with increased concentrations of neutrophil chemoattractants, such as epithelial neutrophil-activating peptide-78 (ENA-78) and IL-8 (CXCL8), in peritoneal fluid and serum of patients with endometriosis, respectively (15, 16).

In a murine endometriosis model study, it was demonstrated that peak neutrophil infiltration occurs in early stages of disease development with subsequent reduction in their numbers, suggesting an important role of neutrophils specifically in initial stages of endometriotic lesion formation (17). Early neutrophil depletion in mice using anti-Gr-1 antibodies led to decreased number and weight of endometriotic lesions, while depletion at late stages produced no such effect (18). Data from Yong-Jin Na et al. complement understanding of neutrophil roles in heterotopia implantation, showing that peripheral blood neutrophils stimulated by peritoneal fluid from endometriosis patients demonstrate increased vascular endothelial growth factor (VEGF) secretion compared to controls (19). These results indicate reduced classical cytotoxic function and potential role of neutrophil-mediated angiogenesis in endometriosis pathogenesis.

Beyond participation in endometriotic lesion initiation, neutrophils may contribute to inflammation chronicity in endometriosis. Patients with endometriosis demonstrate reduced capacity for spontaneous neutrophil apoptosis in both peritoneal fluid and peripheral blood. The extended neutrophil lifespan is associated with antiapoptotic factors in endometriotic peritoneal fluid, including IL-8 and GM-CSF (20). *In vitro* studies have shown that C5a also inhibits neutrophil apoptosis (21, 22). Similar effects are likely mediated by endogenous C5a generated upon complement activation in the peritoneal cavity.

### 2.2 Monocyte-macrophage system

#### 2.2.1 Migration to lesions

One of the key components of innate immunity in the context of endometriosis is the monocyte-macrophage system. Monocytes circulating in blood represent the primary source of tissue macrophages, differentiating into various macrophage subtypes depending on local microenvironment. Endometriosis is characterized by the pathological activation and redistribution of macrophages, contributing to the development of endometriotic lesions. Endometriotic cells can actively recruit monocytes from the bloodstream and attract macrophages differentiating predominantly from monocytes to lesions through production of various chemoattractant. Key factors mediating monocyte/macrophage chemotaxis to endometriotic lesions are chemokines monocyte chemoattractant protein-1 (MCP-1), IL-8, regulated on activation, normal T-cell expressed and secreted (RANTES), and fractalkine (23–25). Specifically, MCP-1 ensures monocyte migration from blood, while IL-8 directs their differentiation toward macrophages with anti-inflammatory M2 phenotype. Fractalkine (CX3CL1), expressed on endometriotic tissue cells, binding to its receptor CX3CR1 on monocytes and macrophages, promotes their adhesion and infiltration in ectopic lesions, and stimulates production of M2-associated cytokines such as IL-10 and TGF-β. Monocyte migration from bloodstream and macrophage accumulation in endometriotic lesions is accompanied by changes in their phenotype and functions, playing a key role in disease pathogenesis.

#### 2.2.2 Macrophage polarization in endometriosis: molecular mechanisms and factors of phenotypic switching

In endometriosis immunopathogenesis, imbalance between classically activated (M1) and alternatively activated (M2) macrophages is noted. It is important to note that systemic monocyte activation can significantly influence the phenotype and functions of macrophages differentiating from them in lesions. Patients with endometriosis exhibit systemic monocyte activation: their spontaneous and induced production of proinflammatory cytokines TNF-α, IL-6, and IL-8 is elevated with normal levels of anti-inflammatory IL-10, indicating a proactive state of the monocyte-macrophage system (7). These mediators can stimulate endometrial cell proliferation, maintain inflammation, angiogenesis, and autoantibody production. Similar changes are found locally: peritoneal macrophages, predominantly originating from recruited monocytes, in endometriosis patients demonstrate signs of shifting toward M1 profile in early disease stages. Thus, in stages I-II, M1 macrophages (CD68^+^) predominate with excessive secretion of IL-6, IL-8, and TNF-α (26–28). Despite increased numbers, these M1 cells have reduced cytotoxic activity against ectopic endometrial cells (29).

With disease progression to stages III-IV, switching of macrophages differentiating from monocytes toward M2 phenotype occurs: the proportion of macrophages expressing CD163 increases, and their IL-10 secretion intensifies (26, 27, 30–32). M2 macrophages become dominant in late stages, corresponding to chronic, protracted inflammation. Classical M2 polarization is mediated by the canonical JAK-STAT6 pathway: IL-4 and IL-13, binding to IL-4Rα, activate JAK kinases and STAT6, initiating transcription of M2 genes (27). In addition to the canonical pathway, switching to M2 is mediated by activation of the Smad2/Smad3 signaling pathway in macrophages under influence of IL-6 and TGF-β produced by endometriotic tissue stromal cells (29, 33, 34). Additionally, epigenetic mechanisms—deoxyribonucleic acid (DNA) methylation and histone modifications—play important roles in regulating macrophage polarization at the level of M2-associated gene transcription: methyltransferases and histone deacetylases promote M2 polarization, while demethylases and acetyltransferases support M1 activation (35). This may be significant for chronic inflammation progression in endometriosis.

Macrophage shifting toward M2 phenotype is associated with chronic stimulation by damage-associated molecular patterns (DAMPs) released from ectopic lesions during cyclical tissue damage and M2-inducing factor secretion (TGF-β, IL-10) by endometriotic cells (30, 34). By analogy with tolerance phenomena during prolonged low-dose lipopolysaccharide (LPS) exposure, it has been shown that under chronic stimulation, macrophages acquire a tolerant, immunosuppressive phenotype (36). Thus, in experimental modeling, low-dose LPS exposure caused increased expression of M2 markers (CD206, CD163) in macrophages and reduced expression of CCR2, CXCR4 receptors and CD80 co-stimulator (32).

**Figure 1:**
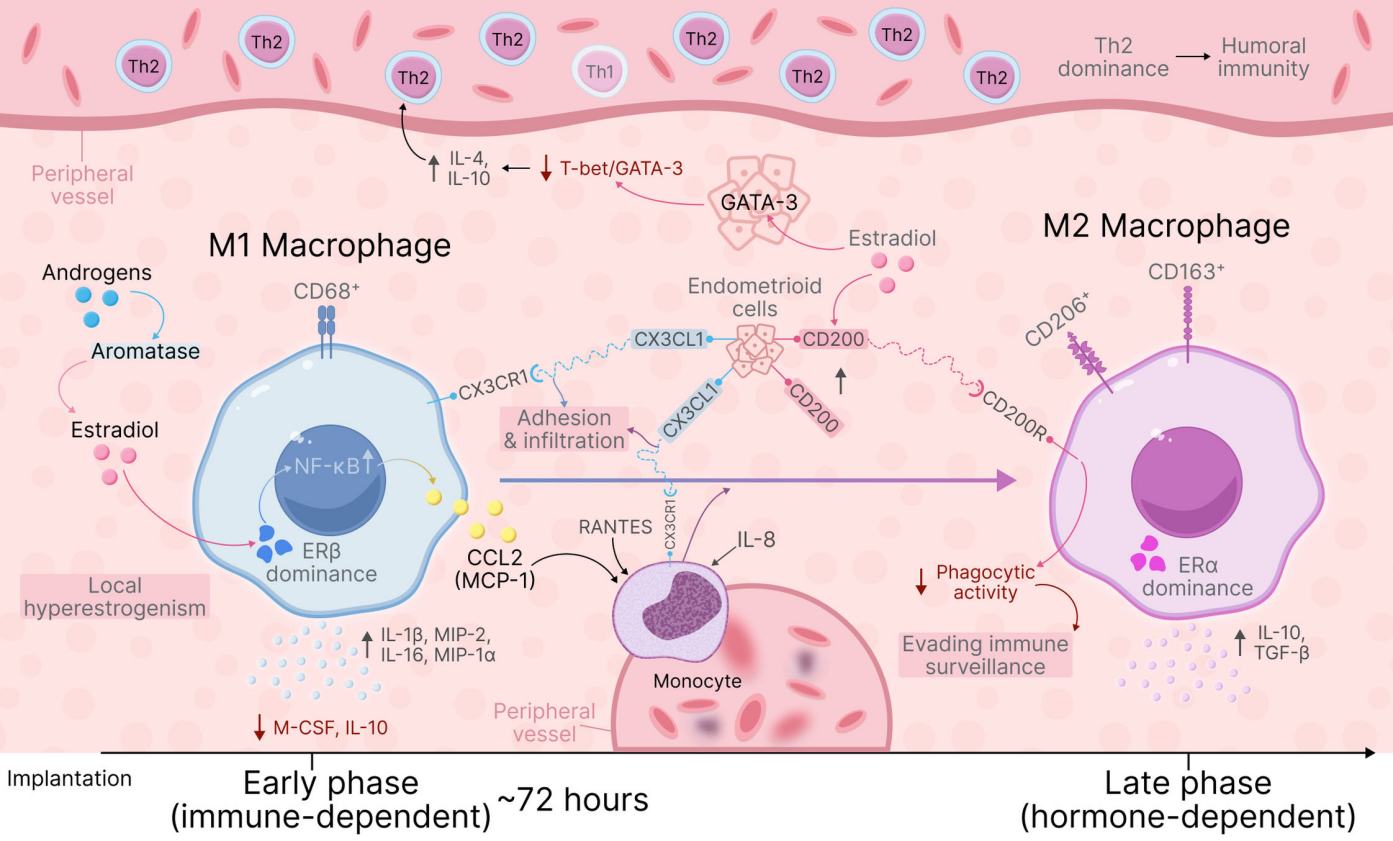
The diagram demonstrates universal hormonal-immune mechanisms operating across all endometriosis forms: local hyperestrogenism via aromatase activation; biphasic immune response with early M1-macrophage phase (ERβ-dominant, 0-72h) producing proinflammatory cytokines, followed by M2-switching (ERα-activation); estradiol-ERβ-NF-κB-CCL2 cascade recruiting monocytes; CD200 upregulation suppressing macrophage phagocytosis; and systemic Th1→Th2 shift via GATA-3 regulation. These mechanisms provide the fundamental estrogen-dependent immunopathogenetic basis for endometriosis regardless of localization.

Sex hormones, particularly estrogens, also significantly influence macrophage functions in endometriosis (**Figure 1**). Endometriotic heterotopias exhibit locally elevated estradiol concentrations due to high aromatase activity converting androgens to estrogens (37, 38). Experimentally, estrogens have been shown to participate in macrophage response switching. In a murine endometriosis model, a biphasic process was identified: in the early immune-dependent phase (first approximately 72 hours after implantation), innate immune response dominates with rapid lesion infiltration by proinflammatory M1 macrophages, predominantly expressing estrogen receptor β (ERβ). Subsequently, the hormone-dependent phase begins, in which ERα expression predominates on macrophages, associated with transition to M2 phenotype (17, 39). Increased ERβ expression in experimental models leads to enhanced production of proinflammatory cytokines (IL-1β, macrophage inflammatory protein-2 [MIP-2], IL-16, MIP-1α) and reduced generation of anti-inflammatory factors (macrophage colony-stimulating factor [M-CSF], IL-10) by macrophages. Meanwhile, ERα activation can partially suppress ERβ-mediated proinflammatory action (for example, by inhibiting excessive inflammasome activation) (40). Furthermore, estrogen receptor activation on macrophages enhances growth factor production (for example, hepatocyte growth factor [HGF]), which in estradiol presence stimulates proliferation of both endometrial stromal cells and macrophages themselves, promoting endometriotic lesion progression (41). However, monocyte numbers in lesions also increase: estradiol-induced ERβ activation increases CCL2 (MCP-1) expression in endometriotic tissue via the nuclear factor kappa B (NF-κB) pathway (37). Under these conditions, monocytes differentiate into M2 macrophages, supplementing the immunosuppressive microenvironment. Simultaneously, enhanced estrogenic stimulation causes hyperexpression of inhibitory molecule CD200 in endometrium, which suppresses macrophage phagocytic activity and thereby helps ectopic cells evade immune surveillance (42).

#### 2.2.3 Macrophages in supporting endometriotic lesion growth

Macrophages not only support endometriotic cell survival but also stimulate their growth and invasion. These cells secrete numerous factors promoting endometriotic tissue proliferation: proinflammatory cytokines (TNF-α, IL-6, IL-8) and growth factors (for example, epidermal growth factor [EGF], VEGF) (24, 43–45). Normally, TNF-α induces apoptosis; however, in the endometriosis context, ectopic cell sensitivity to TNF-α proapoptotic effects is reduced. Conversely, there is evidence that endometriotic cells begin utilizing TNF-α as a survival and growth factor through switching from TNFR1 apoptotic signaling to TNFR2/NF-κB proliferative pathways (46, 47). Similarly, IL-6 secreted by macrophages can enhance endometrial cell proliferation by interacting with the ERα signaling cascade within target cells (17). The persistence of activated macrophages in the lesions is maintained by their resistance to apoptosis: in the peritoneal cavity in endometriosis, macrophages demonstrate increased expression of the anti-apoptotic protein Bcl-2, which prevents their death and allows them to serve as a constant source of TNF-α and other cytokines, thereby enhancing disease progression (32, 44, 48).

Beyond direct growth stimulation, macrophages promote endometriotic cell invasion. M2-like macrophages produce high levels of IL-6, which activates the IL-6/signal transducer and activator of transcription 3 (STAT3) pathway in endometrial cells and increases expression of matrix metalloproteinases MMP-2 and MMP-9 (24). Co-culture of macrophages with endometriotic cells has been shown to lead to enhanced MMP production, especially under hypoxic conditions (through heme oxygenase-1 [HO-1] and TGF-β induction) (43). Increased MMP content promotes migration, invasion, and further growth of endometriotic heterotopias, playing a significant role in endometriosis progression.

Macrophages also actively stimulate angiogenesis and neuroangiogenesis in endometriotic lesions. They release growth factors VEGF, EGF, platelet-derived growth factor (PDGF), and others, promoting new vascular network formation (45). Enhanced vascularization and innervation of lesions create favorable conditions for their further growth and spread. Specifically, co-culture of macrophages with endometrial stromal cells has been shown to substantially increase clonogenicity and invasiveness of the latter through secretion of colony-stimulating factor-1 (CSF-1) and, likely, other undefined factors activating kinase signaling pathways; this interaction is considered a potential therapeutic target (49). Additionally, M2 macrophages can suppress expression of antitumor cytokine IL-24 and its receptor on endometrial stromal cells, thereby enhancing proliferation and invasion of the latter (through increased markers Ki-67, proliferating cell nuclear antigen [PCNA], COX-2, and decreased CD82 suppressor levels)—this mechanism also contributes to endometriosis development (50).

#### 2.2.4 Mechanisms of endometriotic cell immune evasion and macrophage role in suppressing antitumor surveillance

Macrophages in endometriotic lesions contribute to immune evasion by suppressing immune surveillance. M2 cells play a particularly important role here, producing large amounts of immunosuppressive cytokines IL-10 and TGF-β, which suppress cytotoxic lymphocyte activity—NK cells and CD8^+^ T-lymphocytes (51). Such an immunosuppressive effect facilitates the implantation and growth of endometriotic lesions. Additionally, endometriotic stromal cells with high expression of enzyme IDO1 induce a tolerant phenotype in macrophages with reduced phagocytic activity, and these macrophages, in turn, stimulate proliferation and survival of ectopic endometrial cells (52). Recently, it has been demonstrated that exosomes (extracellular vesicles) secreted by ectopic endometrium contain the specific pseudogene LGMNP1, which is taken up by macrophages and causes increased expression of the enzyme legumain (LGMN) in macrophages, promoting their polarization to M2-like phenotype (53). Thus, endometriotic cells through secreted soluble factors and exosomes actively form a “protective” immunosuppressive microenvironment around themselves.

Soluble forms sPD-L1 and sHLA-G, produced by endometriotic cells, also possess immunosuppressive properties, suppressing cytotoxic lymphocytes and promoting immune evasion (54). Notably, sHLA-G plays a similar role during pregnancy, providing immunological tolerance of the maternal organism toward the fetus (55). Furthermore, Fas-mediated apoptosis of immune cells plays an important role: patients with endometriosis show increased expression of Fas (CD95) on peritoneal macrophages and elevated concentration of its soluble form sFas, leading to accelerated macrophage apoptosis (56). Remaining (“surviving”) macrophages secrete PDGF and TGF-β, inducing FasL expression on endometriotic cells and causing death of Fas-positive lymphocytes (57). This dual mechanism (direct macrophage death and indirect lymphocyte elimination) promotes formation of immunologically tolerant phenotype and disease progression. Finally, endometriotic cells secrete soluble form of adhesion molecule intercellular adhesion molecule-1 (ICAM-1), which binds to lymphocyte function-associated antigen-1 (LFA-1) receptor on lymphocytes, preventing their adhesion to lesions and cytolysis of endometriotic cells (58). Excessive sICAM-1 secretion also disrupts immune surveillance in endometriotic lesions.

It should be emphasized that ectopic endometrium in lesions substantially differs from normal endometrium in several parameters: it is characterized by local hyperestrogenism, ERβ hyperexpression, progesterone resistance, and activation of several antiapoptotic pathways (Bcl-2, extracellular signal-regulated kinases 1/2 [ERK1/2], protein kinase B [Akt]) (59). These changes enhance inflammatory response and promote immune control evasion.

The interaction of endometriotic cells, macrophages, and estrogens forms **a vicious cycle** supporting development and growth of endometriotic heterotopias. M2 macrophage predominance and reduced cytotoxic function under estrogen influence leads to ineffective elimination of ectopic endometrial cells, promoting their survival and proliferation. Paracrine interactions between endometriotic cells and macrophages – through secretion of chemoattractants, cytokines, and growth factors – create conditions favorable for endometriosis progression. The imbalance between M1 and M2 macrophages and their functional restructuring in an estrogen-dependent environment are among the key immunopathogenetic mechanisms of endometriosis development.

**Figure 2:**
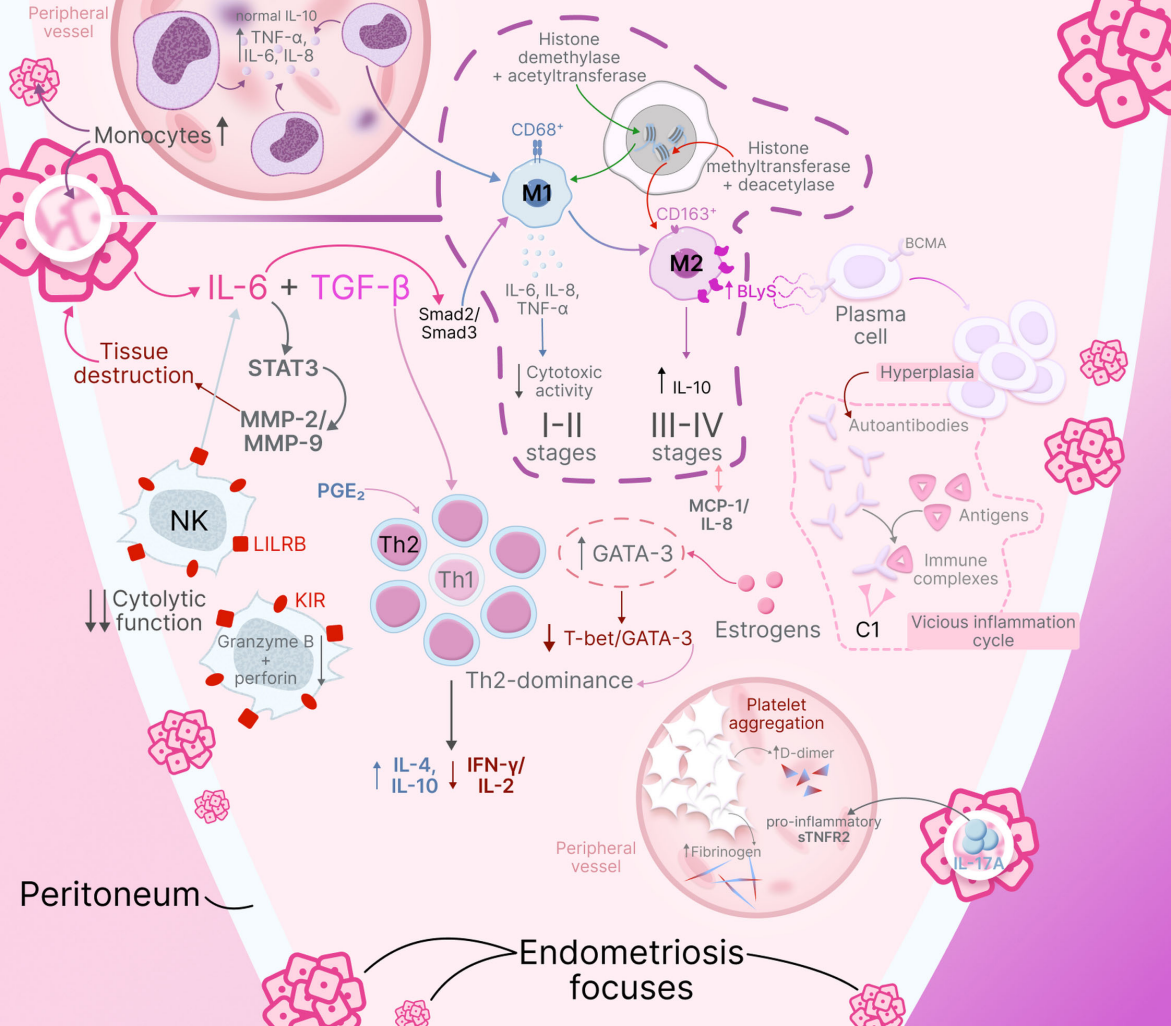
Immunopathogenesis of peritoneal endometriosis. Schematic representation illustrates key immune mechanisms including: systemic monocyte activation with elevated TNF-α, IL-6, and IL-8 production; stage-dependent macrophage switching (stages I-II: CD68^+^ M1 predominance; stages III-IV: CD163^+^ M2 predominance via Smad2/Smad3 pathway); epigenetic regulation of macrophage polarization; NK cell dysfunction with complete inhibition via KIR receptors and IL-6/STAT3/SHP-2 pathway; Th2 dominance driven by estrogens, PGE_2_, and TGF-β; BLyS-mediated B-cell hyperactivation producing multiple autoantibodies; immune complex formation activating complement cascade; and moderate hypercoagulation. The complex cytokine-chemokine network maintains chronic inflammation while creating local immune tolerance.

The immunopathogenetic mechanisms described above – including monocyte-macrophage system activation, phenotypic switching, immune evasion strategies, and suppression of antitumor surveillance—operate in concert to create a unique immunological microenvironment in endometriosis. Peritoneal endometriosis, being the most common and well-studied form of the disease, serves as a paradigmatic model demonstrating the full spectrum of these immune alterations. The complex interplay between innate and adaptive immunity components, hormonal influences, and stage-dependent progression of immunological changes can be comprehensively visualized to illustrate how these multiple pathways converge to support ectopic lesion persistence. (**Figure 2**) provides an integrated schematic representation of the immunopathogenesis of peritoneal endometriosis, synthesizing the key mechanisms discussed in the preceding sections.

### 2.3 Natural killer cells

NK cells are an important component of innate immune response, providing antitumor immune surveillance and protection against viral infections (60). In endometriosis, NK cell dysfunction is discovered, which may contribute to survival, implantation, and proliferation of ectopic endometrial cells (61). Thus, patients with endometriosis show increased expression of inhibitory receptors of the KIR family on NK cell surfaces in peripheral blood and peritoneal fluid (62, 63). It is believed that excessive expression of inhibitory receptors (as well as insufficient expression of activating ones) underlies reduced NK cell ability to recognize and destroy endometriotic cells (64).

As previously mentioned, advanced stages are characterized by local anti-inflammatory microenvironment of endometriotic lesions. Specifically, elevated IL-6 levels in peritoneal fluid induce decreased NK cell cytolytic activity by suppressing granzyme B and perforin expression in these cells—through STAT3 signal activation and Src homology region 2 domain-containing phosphatase-2 (SHP-2) (65). However, the paradoxical nature of this phenomenon lies in the fact that against the background of reduced expression of the key activating receptor NKp46 on peritoneal fluid NK cells, the secretion of the pro-inflammatory cytokines TNF-α and IFN-γ by these cells was increased compared to the control group (66, 67). That is, peritoneal NK cells in endometriosis are less cytotoxic but capable of producing proinflammatory factors, maintaining chronic inflammation. In contrast, the endometrium exhibits a somewhat different picture: TGF-β suppresses IFN-γ production by uterine NK cells (uNK), exerting an inhibitory effect on these cells and creating a local anti-inflammatory microenvironment (68). Blocking endogenous TGF-β in endometrial cell culture leads to increased IFN-γ production, confirming the important regulatory role of TGF-β in uNK activity (69). Additionally, it was established that disease stage influences uNK cell phenotype and functional activity: in early stages, immature cells expressing CD34 and possessing low cytotoxic activity predominate. As disease progresses, the proportion of mature NK cells with CD94 expression increases, characterized by higher functional activity and immune surveillance capacity (70).

Two phenomena contribute to ectopic cell evasion from NK cell cytotoxicity. Soluble forms of NKG2D receptor ligands (MHC class I polypeptide-related sequence A [MICA], MICB, and UL16 binding protein 2 [ULBP-2]), found in elevated concentrations in peritoneal fluid of endometriosis patients, especially in deep infiltrative forms, can bind to NKG2D, causing its internalization and degradation (71). Another mechanism is associated with increased HLA-G molecule expression on endometriotic cells, which also causes inhibition of NK cell cytotoxic activity through interaction with inhibitory receptors immunoglobulin-like transcript 2 (ILT2) and KIR2DL4 (72, 73). Restoring NK cell cytotoxic activity may become a promising therapeutic strategy for endometriosis treatment. Recent studies indicate the possible development of NK-cell exhaustion in endometriosis, similar to that in oncological diseases: in particular, an increased expression of the exhaustion marker PD-1 has been identified on NK-cells in the peritoneal fluid of patients with stages III-IV endometriosis, which allows for the consideration of immune checkpoint inhibitors as a potential therapeutic strategy (74).

### 2.4 Mast cells

Mast cells (MCs) are tissue-resident immune cells whose numbers are significantly elevated in endometriotic lesions compared to eutopic endometrium. Endometriotic lesions create a microenvironment favoring MC recruitment and differentiation through increased expression of stem cell factor (SCF) and genes CPA3, VCAM1, CCL2, CMA1, and KITLG (75). Estrogen enhances MC activation and degranulation, explaining the exacerbation of symptoms during certain phases of the menstrual cycle (76).

Degranulating MCs release histamine, tryptase, TNF-α, IL-6, VEGF, and other mediators that promote inflammation, angiogenesis, fibrosis, and nerve ending sensitization (77). Of particular significance is the close anatomical association between MCs and nerve fibers: in deep infiltrating endometriosis, the number of activated MCs located within 25 μm of nerves is significantly higher than in peritoneal and ovarian forms, which correlates with pain severity (78). Histamine released by MCs activates sensory neurons via the HRH1/TRPV1 pathway, causing hyperalgesia (79). Furthermore, MCs are effectors of the complement system: anaphylatoxin C3a triggers MC degranulation through the C3aR receptor, forming a pro-inflammatory feedback loop (80).

## 3 Adaptive immunity disruptions

Endometriosis is accompanied by pronounced alterations in the adaptive immune system, affecting both T- and B-lymphocytes. These disruptions make significant contributions to disease pathogenesis and may partially explain its chronic progressive course, as well as associations with infertility and pregnancy loss.

### 3.1 T-lymphocytes: Th1/Th2 response, Treg and Th17

One of the key manifestations of adaptive immunity dysregulation in endometriosis is imbalance of T-helper cells type 1 and type 2 (Th1/Th2) (12). Normally, Th1 cells produce proinflammatory cytokines (IFN-γ, TNF-α, IL-2) activating cellular immune response, while Th2 cells secrete cytokines (IL-4, IL-10) stimulating antibody production. In endometriosis, according to most data, a shift of immune response toward Th2 dominance is observed (71). Peritoneal fluid and serum of patients with advanced endometriosis stages show elevated content of Th2-associated cytokines IL-4 and IL-10 and reduced concentration of Th1 cytokines IFN-γ and IL-2 compared to healthy women (12, 71–74). This indicates predominance of humoral immune response. However, understanding Th1/Th2 balance features in endometriosis is complicated by result contradictions: some studies, conversely, note Th1 lymphocyte predominance in patient peripheral blood (14). The immune profile is likely dependent on whether the response is evaluated at the systemic or local level, as well as the disease stage (81). Nevertheless, the overall shift toward Th2 (82, 83) and the associated suppression of local cellular immunity (84)are considered favorable conditions for ectopic tissue persistence.

Factors contributing to polarization toward Th2 phenotype include estrogens, prostaglandin E_2_ (PGE_2_), and TGF-β (12, 85, 86). For example, elevated estradiol levels can shift the balance between Th1 and Th2 through GATA-3 regulation. Thus, excessive transcription factor expression in eutopic endometrium leads to reduced T-bet/GATA-3 ratio and immune response shift toward Th2 (87). TGF-β, produced by macrophages and Treg cells, also promotes Th2 polarization and suppresses Th1 cell differentiation (88).

Along with Th1/Th2 imbalance, regulatory T-lymphocytes (Treg) play important roles in endometriosis. These cells, expressing transcription factor Foxp3 and secreting immunosuppressive cytokines (IL-10, TGF-β), suppress effector T-lymphocyte activity and other immune cells, maintaining tolerance (89). Several studies show increased Treg content in endometriotic lesions: their proportion in peritoneal fluid and endometrium of patients is increased compared to controls (90–92). According to research by Olkowska-Truchanowicz et al., significant elevation of CD25^high^Foxp3^+^ Treg cell proportion in peritoneal fluid and reduction in peripheral blood was revealed in women with stage III-IV ovarian endometriosis compared to controls (87). Authors associate such local Treg elevation in peritoneal fluid with their active recruitment from peripheral blood. Meanwhile, there is evidence of the opposite phenomenon: in some endometriosis forms, deficiency of functionally active Treg lymphocytes is found. Particularly, in deep infiltrative forms, reduced numbers of active Treg cells (CD45RA-Foxp3^hi^) directly in lesions and decreased regulatory (CD4^+^CD25^+^CD103^+^) T-cell numbers in peritoneal cavity were reported (93, 94).

Finally, the Th17 subpopulation participates in endometriosis pathogenesis. The balance of Th17/Treg cells is considered an important factor: shift toward Th17 (producing IL-17) with simultaneous Treg reduction can enhance inflammation (12). There is less definitive data on Th17 role than on Treg function. Recent evidence has elucidated a dysregulated IL-23/Th17 axis in endometriosis, demonstrating that IL-23 drives local immune dysfunction through promotion of Th17 differentiation and IL-17 production, thereby contributing to chronic inflammation and lesion persistence (95).

Recent studies indicate possible development of immune cell exhaustion (T-cell exhaustion) in endometriosis, similar to oncological diseases. Specifically, increased expression of the exhaustion marker PD-1 has been identified on NK cells in peritoneal fluid of patients with stages III-IV endometriosis, allowing consideration of immune checkpoint inhibitors as a potential therapeutic strategy (96).

### 3.2 B-lymphocytes and autoantibodies

B cells also contribute to endometriosis immunopathogenesis, mainly through autoantibody production and immune complex formation. Numerous studies have revealed presence of circulating autoantibodies against various endometrial and ovarian antigens in endometriosis patients (12). So-called antiendometrial antibodies (AEA), detected in patient serum, are considered potential diagnostic markers. It has been shown that primary targets for AEA serve several intracellular components: specifically, tropomyosin-3 (TPM3), stomatin-like protein 2 (SLP2), tropomodulin-3 (TMOD3), α-enolase, serine/threonine protein kinase, and syntaxin-5 (97). According to Mathur et al., transferrin may also serve as an antigen against which AEA are formed (i.e., antibodies to transferrin are detected) in some patients (98). Combined sensitivity of detecting various autoantibodies reaches approximately 78% with 89-96% specificity, significantly exceeding diagnostic value of traditional marker cancer antigen 125 (CA-125) (99). According to a multicenter study involving 2609 patients, indirect immunofluorescence for AEA detection demonstrated high accuracy (sensitivity approximately 87%, specificity 87%) compared to laparoscopy results, allowing recommendation as effective screening test before surgical intervention (97).

Besides antiendometrial antibodies, patients with endometriosis frequently show antiovarian antibodies, antinuclear antibodies, and autoantibodies to laminin-1 (100). In endometriosis patients, antibodies to laminin-1 (aLN-1) may disrupt implantation and placentation processes, explaining endometriosis association with infertility and reproductive failures in *in vitro* fertilization (101). Formation of such autoantibody spectrum is likely associated with loss of immunological tolerance to self-antigens, molecular mimicry phenomena, or appearance of modified autoantigens due to inflammation and tissue destruction. Inflammation and tissue destruction are also potentiated by immune complexes formed with endometrial cells, which initiate classical complement pathway activation through C1 complex, forming a vicious cycle of disease progression (102).

An important feature of humoral immunity in endometriosis is that autoantibody production is controlled predominantly by Th2 cytokines. Elevated IL-4 and IL-10 levels stimulate B-lymphocyte proliferation and their differentiation into plasma cells (6). Estradiol also plays a role: it regulates GATA3 expression in endometrial epithelial cells, thereby enhancing their production of anti-inflammatory cytokines (IL-4, IL-10) (103). This, in turn, maintains humoral immunity activation. Moreover, in endometriosis, increased expression of B-lymphocyte stimulator (BLyS, also known as BAFF) by macrophages in the lesions has been detected. This leads to the hyperstimulation and prolonged survival of plasma cells through interaction with the BCMA receptor on them (104).

The combination of Th2/Treg response predominance with B-cell hyperactivation leads to frequent realization of autoimmune process features in endometriosis. On one hand, local immunosuppression (Th2 and Treg) promotes lesion evasion from cytotoxic immunity; on the other hand, B-lymphocyte stimulation leads to autoantibody production maintaining chronic inflammation. The complex nature of such disruptions is confirmed by frequent association of endometriosis with other autoimmune diseases and immune balance disorders (105).

## 4 Cytokine profile and growth factors in endometriosis

Endometriosis is characterized by complex alterations in cytokine profiles—signaling molecules mediating interactions between immune system cells and endometrium. Imbalances of both proinflammatory and anti-inflammatory cytokines are noted, as well as levels of various growth factors and chemokines in blood serum and, especially, peritoneal fluid, collectively forming a special inflammatory-immune microenvironment (106). The main sources of these cytokines are activated peritoneal macrophages, monocytes recruited from peripheral blood, endometrial stromal cells, and endometriotic cells themselves.

### 4.1 Proinflammatory cytokines

Patients with endometriosis show elevated concentrations in peritoneal fluid of key cytokines supporting cell-mediated immunity and inflammation: IL-1β, IL-6, and TNF-α (107, 108). Collectively, proinflammatory cytokines participate in local inflammatory reaction and promote estrogen-dependent endometriotic tissue growth—through aromatase induction and altered steroid hormone receptor expression (109, 110). The study by Drosdzol-Cop et al. showed significant elevation of IL-6, TNF-α, and glycodelin A levels in peritoneal fluid of endometriosis patients, with TNF-α demonstrating greatest diagnostic value (sensitivity 81.8%, specificity 76.5%), although no statistically significant correlation between cytokine levels and disease stage was found, consistent with previously published review by Harada et al. (111, 112). However, other authors demonstrated statistical significance of correlations between proinflammatory cytokine levels in peritoneal fluid and disease severity (113–115).

An important feature of proinflammatory cytokines in endometriosis is their predominant orientation toward stimulating angiogenesis and proliferation rather than classical inflammatory response (116). Thus, IL-1β and TNF-α, produced by activated macrophages, stimulate angiogenic factor secretion and promote adhesion of endometrial stromal cells to mesothelium, respectively (117, 118). While IL-6, normally inhibiting endometrial cell proliferation, paradoxically contributes to uncontrolled growth of endometriotic lesions due to development of resistance to its antiproliferative action (119).

Proinflammatory cytokine levels are significantly higher in peritoneal fluid compared to patient serum, indicating predominantly local nature of their production by activated peritoneal macrophages, recruited peripheral blood monocytes, and autocrine secretion by endometriotic cells (120, 121). Recent studies have shown increased expression of IL-17A in ectopic endometriosis lesions, which stimulates the production of angiogenic and pro-inflammatory factors, thereby promoting the maintenance of endometriotic lesions (122).

### 4.2 Anti-inflammatory cytokines

Besides proinflammatory cytokines, endometriosis patients show elevated levels of immunoregulatory cytokines IL-10 and TGF-β (34, 123). The latter serve as main mediators of immune “privileged” status formation in endometriotic lesions. Increased concentrations in endometriosis can be considered compensation for chronic inflammation and organism’s attempt to limit tissue damage. On the other hand, excessive IL-10 and TGF-β lead to immune evasion of ectopic tissue—reduced antitumor surveillance (86, 88). TGF-β not only suppresses Th1 response but also stimulates endometrial stromal cell invasion and fibrosis development in lesions (124). Ultimately, balance shifts toward immune tolerance, favoring heterotopia persistence.

### 4.3 Chemokines and growth factors

Endometriosis is accompanied by activation of secretion of numerous chemokines attracting immune cells, as well as growth factors ensuring neoangiogenesis. Peritoneal fluid and plasma of patients show elevated concentrations of MCP-1 (CCL2) and IL-8 (CXCL8), which are powerful attractants for monocytes, macrophages, and neutrophils (125, 126). It has been established that MCP-1 and IL-8 levels in peritoneal fluid correlate with disease stage: in more severe endometriosis, their concentrations are higher (127).

VEGF is a key angiogenesis stimulator and is elevated both locally (in peritoneal fluid and endometriotic lesions) and systemically in endometriosis patients (128, 129). VEGF is secreted by activated macrophages and endometrial cells under influence of proinflammatory cytokines and estrogens (130). Enhanced angiogenesis promotes vascularization and growth of endometriotic implants.

Overall, the cytokine-chemokine profile in endometriosis is shifted toward maintaining chronic inflammation, attracting immune cells, and stimulating angiogenesis. At the systemic level in patient blood, elevated levels of IL-1β and soluble TNF receptor type 2 (sTNFR2) are frequently recorded, with the latter considered a potential early marker of endometriosis development. It is noted that such changes are predominantly expressed in relatively young patients (<40 years) (131). At the same time, it is important to note that individual cytokines and chemokines have limited diagnostic value. However, their combination in multimarker panels (including VEGF, MCP-1, and CA-125) achieves high diagnostic accuracy (sensitivity of 81-93%) in the non-invasive diagnosis of endometriosis, particularly when the phase of the menstrual cycle is taken into account (132, 133).

Thus, immune environment in endometriosis is characterized by simultaneous activation of proinflammatory cytokines (maintaining pathological inflammation and lesion growth) and compensatory elevation of anti-inflammatory factors (creating local tolerance to endometriotic tissues). Cytokine imbalance ensures both chronic inflammation damaging tissues and immunosuppression preventing elimination of ectopic implants. This dynamic equilibrium differs at various stages and in different disease forms, reflecting complexity of endometriosis immunopathogenesis.

## 5 Role of platelets in endometriosis

Recent years have provided data on important platelet participation in endometriosis pathogenesis. Platelets are traditionally known for their role in hemostasis; however, in endometriosis they become involved in pathological inflammatory processes, promoting angiogenesis and fibrosis in lesions. The study by Ding et al. confirmed the hypothesis of hypercoagulation in endometriosis. Patients with endometriotic ovarian cysts show signs of coagulation cascade activation: increased platelet aggregation and elevated coagulation markers (D-dimer, soluble P-selectin, prothrombin fragments 1 + 2) with simultaneous thrombin time shortening. Surgical removal of endometriomas led to normalization of these hemostatic parameters (134). Thus, endometriosis is accompanied by hypercoagulation state, likely due to constant minor tissue damage and coagulation factor release.

According to Zhang et al., activated platelets attracted to inflammation and tissue damage sites stimulate endometriosis progression through TGF-β1 secretion and TGF-β/Smad signaling pathway activation. This has been shown to lead to epithelial-mesenchymal transition (EMT) in endometriotic epithelial cells, evidenced by increased numbers of cells expressing mesenchymal marker vimentin, as well as fibroblast-to-myofibroblast differentiation (increased numbers of α-smooth muscle actin [α-SMA]-positive cells). Collectively, these changes enhance cellular contractile activity, collagen production, and lead to fibrosis development and smooth muscle metaplasia of stroma in endometriotic lesions (135, 136). Thus, platelets, accumulating in lesions, promote fibrosis and sclerosis of surrounding tissues, which may contribute to chronic pain syndrome and disease progression.

Experimental work by Guo et al. convincingly demonstrated platelet participation in endometriosis pathogenesis and indicated new therapeutic possibilities. Blocking platelet P-selectin (key platelet adhesion molecule) led to reduced endometriotic implant growth in laboratory animals (137). This suggests that antiplatelet therapy may be considered a potential approach to endometriosis treatment. Specifically, targeting platelet-endometriotic tissue interactions (for example, using selectin inhibitors or other antiplatelet agents) can interrupt the pathological “inflammation-coagulation-fibrosis” cycle in lesions. This approach still requires clinical investigation but represents interest as a non-hormonal method of disease intervention.

## 6 The complement system in the pathogenesis of endometriosis

The complement system represents a critical component of innate immunity, whose role in the pathogenesis of endometriosis has long remained underestimated. This cascade of plasma and membrane-bound proteins performs multiple functions: opsonization of pathogens, chemotaxis of immune cells, direct destruction through the membrane attack complex (MAC), and modulation of inflammatory responses (138). Since the late 1980s, substantial evidence has accumulated indicating the central role of complement in the development and progression of endometriosis through mechanisms of inflammation, angiogenesis, and microbiota interaction (139).

### 6.1 Evolution of knowledge about complement in endometriosis

The first indications of complement system involvement in the pathogenesis of endometriosis appeared in 1987, when Zeller and colleagues discovered increased chemiluminescent activity of monocytes and peritoneal macrophages in women with endometriosis (140). This indirectly indicated complement activation as one of the mechanisms of enhanced activation of innate immune cells and became an early sign of the role of this system in the disease.

Direct evidence of complement participation was obtained in 1989, when Isaacson and colleagues first demonstrated that cultured endometriotic cells independently synthesize and secrete component C3 (139). This discovery fundamentally changed the understanding of immunopathogenesis, showing that endometriotic tissue does not simply passively undergo the action of circulating complement, but actively participates in creating a pro-inflammatory microenvironment through autonomous production of complement components. In 1992, D’Cruz and Wild conducted a comprehensive assessment of complement activation, investigating the specificity of this process for endometrial tissue in endometriosis (141).

Modern proteomic and transcriptomic studies have revealed differential expression of multiple complement components in endometriotic tissues: C3, C4A, C7, factor D (CFD), factor B (CFB), factor H (CFH) (142). These data indicate activation of both classical and alternative complement pathways in the pathogenesis of the disease. Scheerer and colleagues in 2016, while characterizing endometriosis-associated immune cell infiltrates (EMaICI), also found signs of complement activation as part of the complex immune microenvironment of lesions (10).

### 6.2 Pro-inflammatory role of the complement system

Activation of the complement cascade in endometriosis generates powerful pro-inflammatory mediators—anaphylatoxins C3a and C5a—which bind to specific receptors (C3aR and C5aR1/CD88) on immune cells, triggering intense inflammatory reactions (142, 143). In the context of endometriosis, C5a stimulates peritoneal macrophages to produce pro-inflammatory cytokines IL-1β, IL-6, TNF-α, and IL-8, enhancing the cytokine imbalance characteristic of this disease (Section 4) (107, 108).

Studies by Kabut and colleagues showed statistically significantly elevated levels of C3c, C4, and SC5b-9 in peritoneal fluid and serum of women with endometriosis with reduced iC3b levels, and stage-dependent dynamics were revealed: higher iC3b concentrations are characteristic of early stages (I-II), whereas elevated SC5b-9 levels are characteristic of advanced stages (III-IV) (144).

Formation of the membrane attack complex (MAC, C5b-9) on endometriotic cells paradoxically leads not to lysis but to cell activation (142, 144). At sublytic concentrations, MAC induces calcium influx and activates NF-κB and MAPK signaling cascades, resulting in increased production of pro-inflammatory cytokines, adhesion molecules (ICAM-1, VCAM-1), and survival factors (58, 142). Multi-omics analysis revealed a positive correlation between expression of central complement factors (C1S, C1QA, C1R, C3) and tissue factor (TF), indicating cross-interaction between complement and coagulation systems in the pathogenesis of endometriosis (145). These mechanisms ensure persistence of endometriotic cells under immune attack and facilitate their attachment to peritoneal mesothelium—a critical stage in lesion formation.

Beyond direct pro-inflammatory effects, the complement system interacts with the humoral autoimmune component of endometriosis. Sikora and colleagues identified elevated levels of C1q and C1 inhibitor in peritoneal fluid, with higher concentrations in early disease stages (102). These data, along with the stage-dependent dynamics of iC3b and SC5b-9 (144), indicate predominant activation of the classical pathway in the initial phases of the disease. The mechanism is associated with C1q recognition of immune complexes formed by autoantibodies against endometrial antigens (Section 3.2), which integrates B-cell dysfunction with complement-mediated inflammation (97–102).

### 6.3 Pro-angiogenic activity of complement

Beyond pro-inflammatory effects, the complement system plays a critical role in angiogenesis of endometriotic lesions. Component C1q, beyond its canonical function in classical pathway activation, participates in tumor growth, placentation, wound healing, and angiogenesis.

Transcriptomic analysis revealed increased expression of all three C1q genes (C1QA, C1QB, C1QC) in all endometriosis histotypes compared to healthy endometrium, with correlation between expression level and disease severity (146). Immunohistochemically, C1q protein is localized predominantly around vessels in lesions, with CD68+ macrophages being its producers.

Experiments with siRNA silencing of gC1qR (C1q component receptor) confirmed that C1q-gC1qR interaction is critically important for angiogenic functions (146). This mechanism acts synergistically with VEGF-mediated angiogenesis, creating a powerful pro-angiogenic impulse (128, 129). C1q is present in endometriotic lesions and in normal ovarian tissue, but in endometriosis this physiological process acquires a pathological character, supporting the growth and vascularization of ectopic foci.

In addition to C1q, anaphylatoxins C3a and C5a substantially contribute to angiogenesis of endometriotic lesions. They exert pronounced effects on endothelial cells, stimulating production of pro-inflammatory cytokines and chemokines (IL-8, IL-1β, RANTES) through activation of the MAPK/ERK1/2 signaling cascade mediated by G-protein-coupled receptors C3aR and C5aR (147). In ovarian cancer models, genetic deletion or pharmacological blockade of C5aR1 led to reduced endothelial cell activation and impaired neovascularization, while activation of C3aR and C5aR1 on immune and stromal cells stimulated angiogenesis through VEGF-dependent pathways (148).

### 6.4 Interaction of complement with endometrial microbiota

Studies have revealed bidirectional interactions between the complement system and endometrial microbiota in endometriosis. In women with endometriosis, particularly with adenomyosis, microbiotic dysbiosis (increased Escherichia coli, Shigella, Streptococcus, and decreased Lactobacillus) is associated with complement activation (149).

Bacterial components (LPS, peptidoglycans, lipoteichoic acid) activate complement through alternative and lectin pathways. Generation of C3a and C5a enhances inflammation, while sublytic MAC formation increases expression of adhesion molecules (ICAM-1, VCAM-1, E-selectin) on endometrial cells, facilitating their attachment to peritoneal mesothelium (58). During phagocytosis of complement-opsonized bacteria, peritoneal macrophages paradoxically polarize toward the M2 phenotype through CR3, producing IL-10 and TGF-β while suppressing IL-12 and TNF-α (30, 34, 51). Dysbiotic microbiota also reduces expression of membrane complement inhibitors (CD46, CD55, CD59) through TLR signaling, enhancing cellular susceptibility to chronic inflammatory stress (149).

### 6.5 Integration of complement into the overall immunopathogenetic picture

The complement system occupies a central position in the immunopathogenesis of endometriosis, integrating multiple pathological processes. It links innate immunity (activation of macrophages and neutrophils, Sections 2.1 and 2.2) with adaptive immunity (formation of immune complexes with autoantibodies, Section 3.2), enhances cytokine imbalance (Section 4), stimulates angiogenesis (Section 4.3), and interacts with the inflammation-coagulation platform (Section 5).

It is important to note the stage-dependent dynamics of complement activation: in early stages (I-II), an acute inflammatory phase predominates with anaphylatoxin generation and M1 macrophage recruitment, whereas in late stages (III-IV), complement activation contributes to formation of an immunosuppressive microenvironment through induction of M2 polarization and Treg expansion (26–28, 90–92).

Different forms of endometriosis are characterized by specific features of complement system activation. In ovarian endometriosis (endometriomas), local C3 production by glandular epithelial cells lining the inner surface of cysts has been identified; expression of genes C1R, C3, and C7 is elevated in ectopic tissue compared to eutopic endometrium (80, 150). Additionally, the presence of C1q in ovarian endometriomas is associated with pro-angiogenic functions independent of the classical cascade activation pathway (146).

In deep infiltrating endometriosis (DIE), the greatest number of activated and degranulating mast cells closely associated with nerve endings is observed (130). Since C3a is a potent stimulus for mast cell activation through C3aR, enhanced complement-dependent degranulation in DIE explains the more pronounced pain syndrome in this form of the disease (80).

In peritoneal endometriosis, multi-omics analysis demonstrated hyperexpression of C3, C7, and C1QA in ectopic lesions compared to eutopic endometrium, with the complement and coagulation pathway being the most enriched KEGG signaling pathway in this localization (151).

## 7 Immune microenvironment features in different forms of endometriosis

Endometriosis is a heterogeneous disease, and immunopathogenesis may somewhat differ depending on lesion localization. External (superficial) forms include peritoneal and ovarian endometriosis, as well as deep infiltrative endometriosis (rectovesical pouch involvement, rectocervical). Internal endometriosis represents adenomyosis (myometrium involvement). Extragenital forms of endometriosis are less common—involving intestine, urinary bladder, surgical scars, and other organs (10). Immune microenvironment in different endometriosis forms is actively studied, as local immunity features are believed to influence clinical course and disease progression.

It should be noted that immunological profile has been most completely studied in ovarian endometriosis, rectocervical (rectovaginal) endometriosis (particular case of deep infiltrative), and extragenital forms, since these variants usually require surgical treatment and provide sufficient material for research (152). Adenomyosis in early stages often responds to conservative therapy, and histological examination (after hysterectomy) is performed mainly in severe resistant cases, somewhat limiting adenomyosis immunopathogenesis data.

### 7.1 Ovarian endometriosis

Endometriotic ovarian cysts (endometriomas) are one of the most common endometriosis forms, occurring in 17-44% of patients with this disease according to various data (153). Ovarian endometriosis is characterized by pronounced both local and systemic immune changes, which collectively promote ectopic lesion implantation and growth.

The formation of an immunosuppressive microenvironment in endometriomas is associated with the expression of the pseudogene LGMNP1, which is involved in M2 macrophage polarization (57). These macrophages, expressing the surface markers CD163^+^ and CD206^+^, participate in the vascularization of endometriotic lesions (154). Along with vascular network, innervation plays important roles in endometrioma maintenance. Mast cell interaction with nerve fibers has been shown to lead to cystic wall neoinervation, potentially explaining pain syndrome formation mechanisms in this disease form (78). Additionally, endometriomas show increased Treg content and reduced NK cell cytotoxic activity, promoting ectopic cell evasion from immune surveillance (51, 88).

Peritoneal fluid in ovarian endometriosis also has characteristic profile. Endometriotic stromal cell interaction with macrophages leads to enhanced MCP-1, IL-1β, IL-6, IL-8, TNF-α production, creating self-sustaining inflammatory cascade (154). Moreover, IL-8 level correlates with disease severity, and IL-6 level also demonstrates association with adhesive process (16, 155). Chronic inflammation in abdominal cavity in ovarian endometriosis is accompanied by hemostatic system activation: such patients show elevated D-dimer, fibrinogen, and fibrin degradation product levels, indicating close connection between inflammatory and coagulation processes.

**Figure 3:**
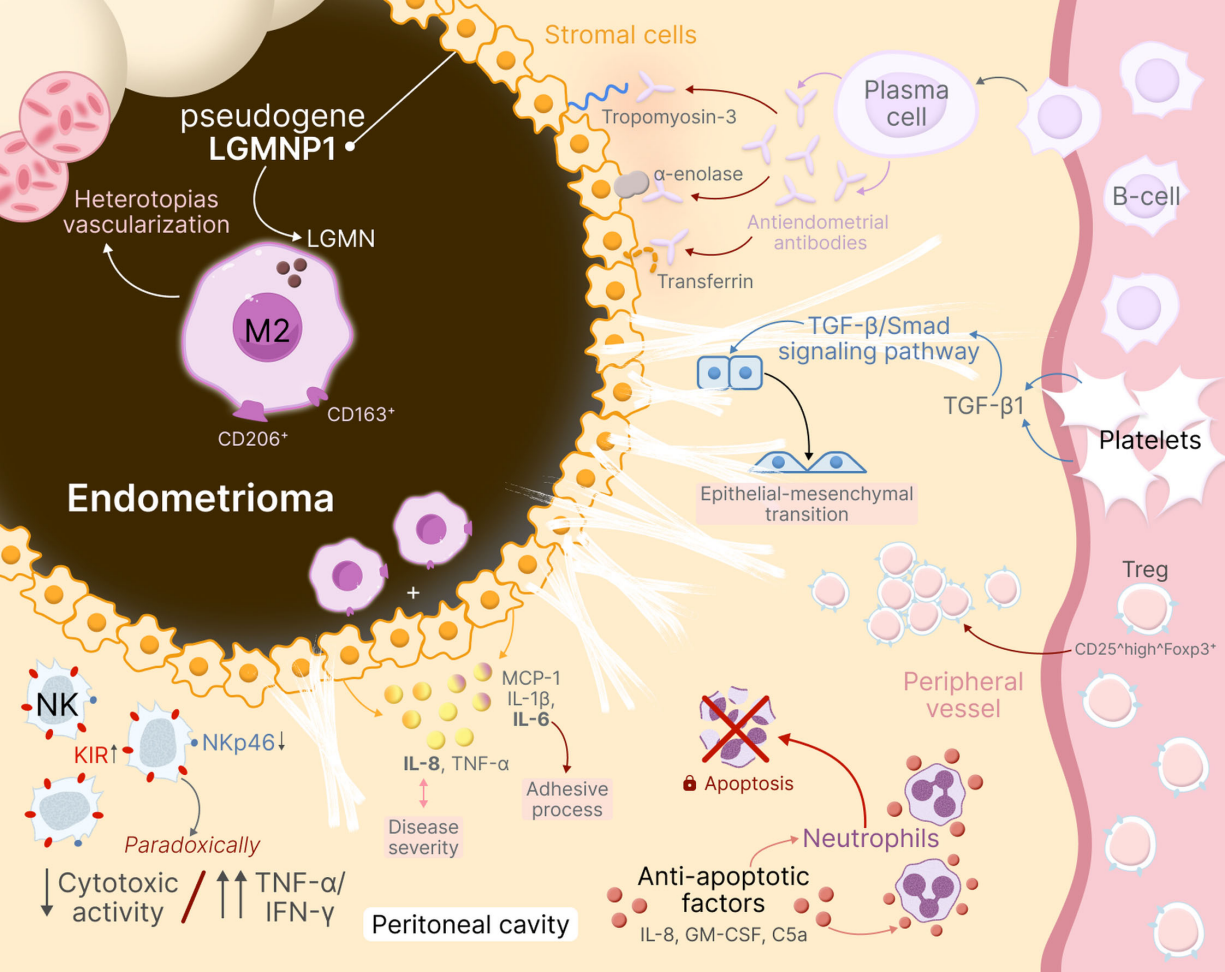
Immunopathogenesis of ovarian endometriosis (endometriomas). The diagram demonstrates: LGMNP1 pseudogene expression inducing M2 macrophage polarization (CD163^+^/CD206^+^) and lesion vascularization; neutrophil resistance to apoptosis mediated by IL-8, GM-CSF, and C5a with prolonged lifespan and VEGF secretion; NK cell dysfunction with increased KIR inhibitory receptors and decreased NKp46, resulting in reduced cytotoxicity but preserved TNF-α/IFN-γ production; CD25^high^Foxp3^+^ Treg migration from peripheral blood into peritoneal cavity; B-cell activation producing autoantibodies against tropomyosin-3, α-enolase, and transferrin (78% sensitivity); cytokine network in peritoneal fluid (MCP-1, IL-1β, IL-6, IL-8, TNF-α) with IL-8 correlating with disease severity; and platelet activation initiating TGF-β1/Smad signaling leading to epithelial-mesenchymal transition and early fibrosis.

Overall, the immunological profile of ovarian endometriosis can be characterized by local immunosuppression (antitumor immunity suppression in cysts due to excess Treg, M2, and reduced NK functions) combined with systemic inflammatory response (elevated proinflammatory cytokines and coagulation activation). Such combination promotes disease progression: on one hand, immunity does not destroy lesions; on the other hand, constant inflammation maintains their growth (**Figure 3**).

### 7.2 Adenomyosis

Adenomyosis is a form of endometriosis involving invasion of endometrial glands and stroma into myometrium thickness (uterine muscle layer) with diffuse uterine enlargement. Adenomyosis prevalence reaches 20-35% among reproductive-age women (156). Adenomyosis frequently combines with external endometriosis and uterine fibroids, complicating diagnosis and treatment (157).

Immune mechanisms in adenomyosis play important roles, mainly associated with proinflammatory and anti-inflammatory factor imbalance in endometrial and myometrial tissues. On one hand, adenomyosis lesions show increased infiltration by CD163^+^ M2 macrophages. These macrophages secrete various mediators promoting tissue remodeling and lesion growth, including matrix metalloproteinases MMP-2 and MMP-9, PGE_2_, and VEGF (158, 159). MMP release leads to extracellular matrix degradation, facilitating endometrial structure invasion into myometrium, while PGE_2_ and VEGF stimulate neoangiogenesis and smooth muscle cell proliferation, causing myometrial hypertrophy around lesions.

On the other hand, adenomyosis shows signs of immune surveillance deficiency by T-lymphocytes. The study by Gui et al. showed significant reduction in Foxp3^+^ Treg cell density in adenomyosis lesions and peripheral blood of these patients compared to healthy individuals, simultaneously revealing increased Th17/Treg ratio (96). Insufficient immunosuppressive Treg in adenomyosis leads to enhanced local inflammation. This manifests, particularly, as innate immune pathway activation: increased Toll-like receptor 4 (TLR4) expression in endometrial stromal cells in adenomyosis has been shown. Stimulating these cells with bacterial LPS leads to enhanced proliferative activity and proinflammatory cytokine secretion (160). Thus, roles of microbial factors or endogenous TLR ligands (for example, damaged molecules) in provoking inflammation in adenomyosis are suggested.

Nevertheless, the organism attempts to compensate excessive inflammation in adenomyosis. Increased anti-inflammatory IL-10 expression in endometrium is observed, especially pronounced in secretory phase of menstrual cycle (161). Local IL-10 release creates immunosuppressive environment, limiting excessive tissue damage. Notably, the most pronounced immune changes in both adenomyosis and external endometriosis occur specifically in secretory (luteal) phase. During this period, high mobility group box 1 (HMGB-1) levels (cellular damage marker) sharply increase in endometrium of women with endometriosis. Extracellular HMGB-1, released during oxidative stress, interacts with TLR4 on endometrial stromal cells, activating NF-κB and enhancing endometrial cell proliferation and inflammatory factor production (for example, IL-6) (162). This serves as one mechanism of endometriosis development under chronic inflammation influence. Besides HMGB-1, increased fractalkine (CX3CL1) chemokine expression is found in adenomyosis endometrium, attracting immune cells (monocytes, macrophages, NK and T cells) through CX3CR1 receptor (163). Fractalkine maintains inflammation persistence and stimulates proinflammatory cytokine and growth factor production, leading to abnormal endometrial cell proliferation in adenomyosis.

**Figure 4:**
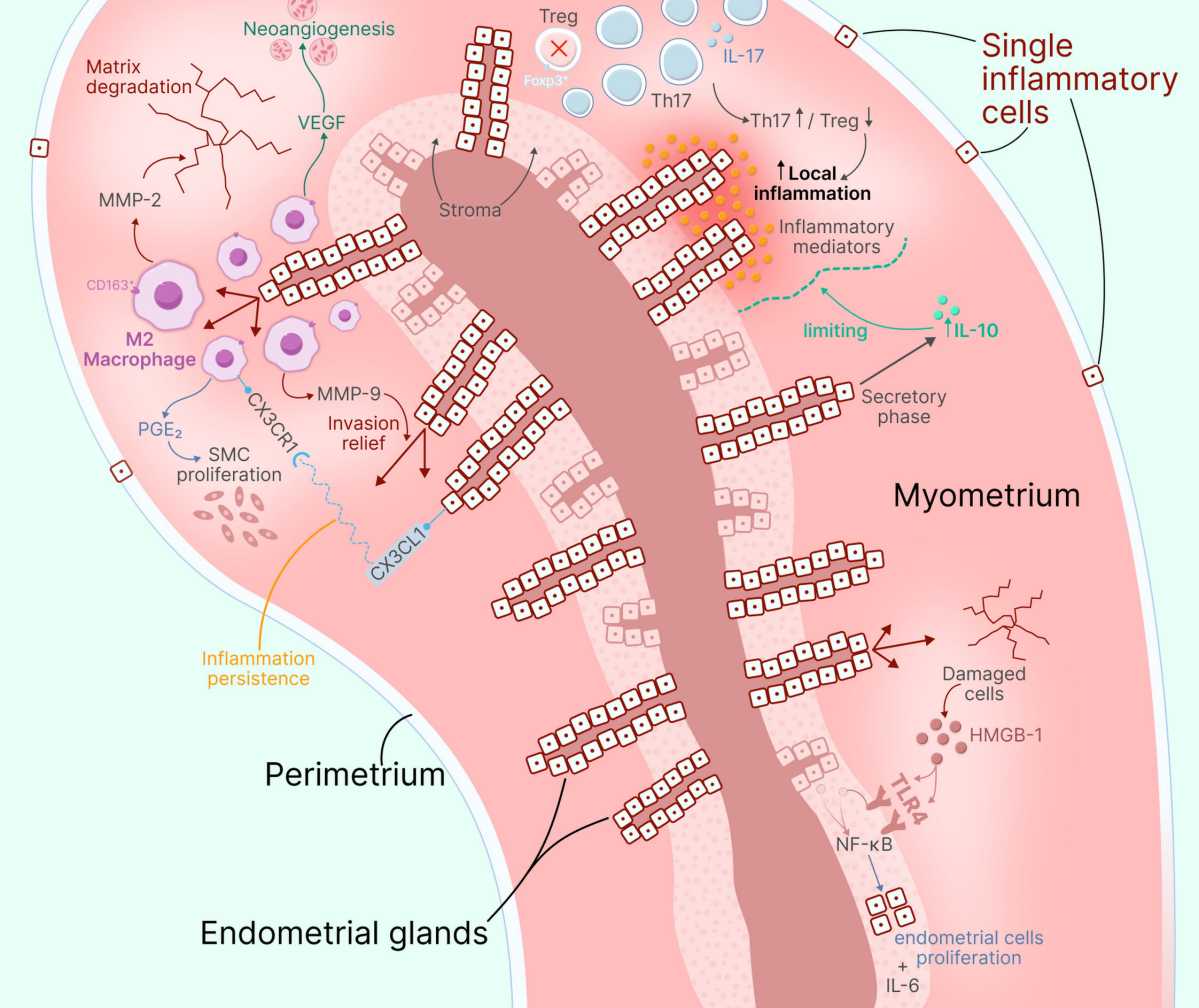
Immunopathogenesis of adenomyosis. The diagram illustrates: CD163^+^ M2 macrophages scattered around invasive endometrial glands in myometrium, secreting MMP-2 and MMP-9 (matrix degradation), PGE_2_ (smooth muscle proliferation), and VEGF (neoangiogenesis); Treg/Th17 imbalance with Foxp3^+^ Treg deficiency and Th17 elevation enhancing local inflammation; TLR4 signaling pathway activation leading to NF-κB-mediated IL-6 production; HMGB-1 cascade (released from damaged cells) triggering TLR4→NF-κB→endometrial cell proliferation and IL-6 production; compensatory IL-10 elevation in secretory phase limiting inflammation; and fractalkine (CX3CL1)/CX3CR1 axis attracting immune cells (monocytes, macrophages, NK, T cells) and maintaining inflammation persistence. Minimal peritoneal changes are shown.

Overall, adenomyosis immunological profile is characterized by pronounced proinflammatory changes, including TLR4 signaling pathway activation and reduced immunosuppressive Treg cells, while M2 macrophages enhance pathological process through secretion of tissue remodeling and angiogenesis factors, despite presence of some compensatory mechanisms in the form of increased IL-10 (**Figure 4**).

### 7.3 Deep infiltrative endometriosis

Deep infiltrative endometriosis is a severe disease form involving endometriotic lesion penetration >5 mm beneath peritoneum. Rectocervical endometriosis (RCE) is a common variant of deep endometriosis, affecting rectovaginal septum, uterosacral ligaments, and rectocervical pouch peritoneum. Clinically, RCE is characterized by pronounced pain syndrome. In prospective study by Donnez et al., including 500 patients with deep endometriosis, rectocervical lesions were associated with extremely high chronic pelvic pain frequency—dysmenorrhea occurred in 95% of patients, dyspareunia in 86.4%, painful defecation (dyschezia) in 48.2% (164). According to Gerasimov et al., among hospitalized patients, rectocervical endometriosis proportion was approximately 20.1% based on surgical protocol analysis (i.e., occurs in every fifth endometriosis patient admitted for surgical treatment) (165).

In rectocervical endometriosis lesions, increased IDO1 expression is associated with increased COX-2 and MMP-9 expression, promoting enhanced adhesion and invasion of endometrial stromal cells (166). This mechanism underlies tissue destruction, oxidative stress occurrence [markers determined in peritoneal fluid (167)], and heterotopia penetration into ligament thickness. Proliferation and heterotopia volume increase are promoted by tolerogenic microenvironment in lesions, arising from interaction between endometrial stromal cells and macrophages and leading to Treg recruitment through CCL17 and CCL22 chemokines (168).

**Figure 5:**
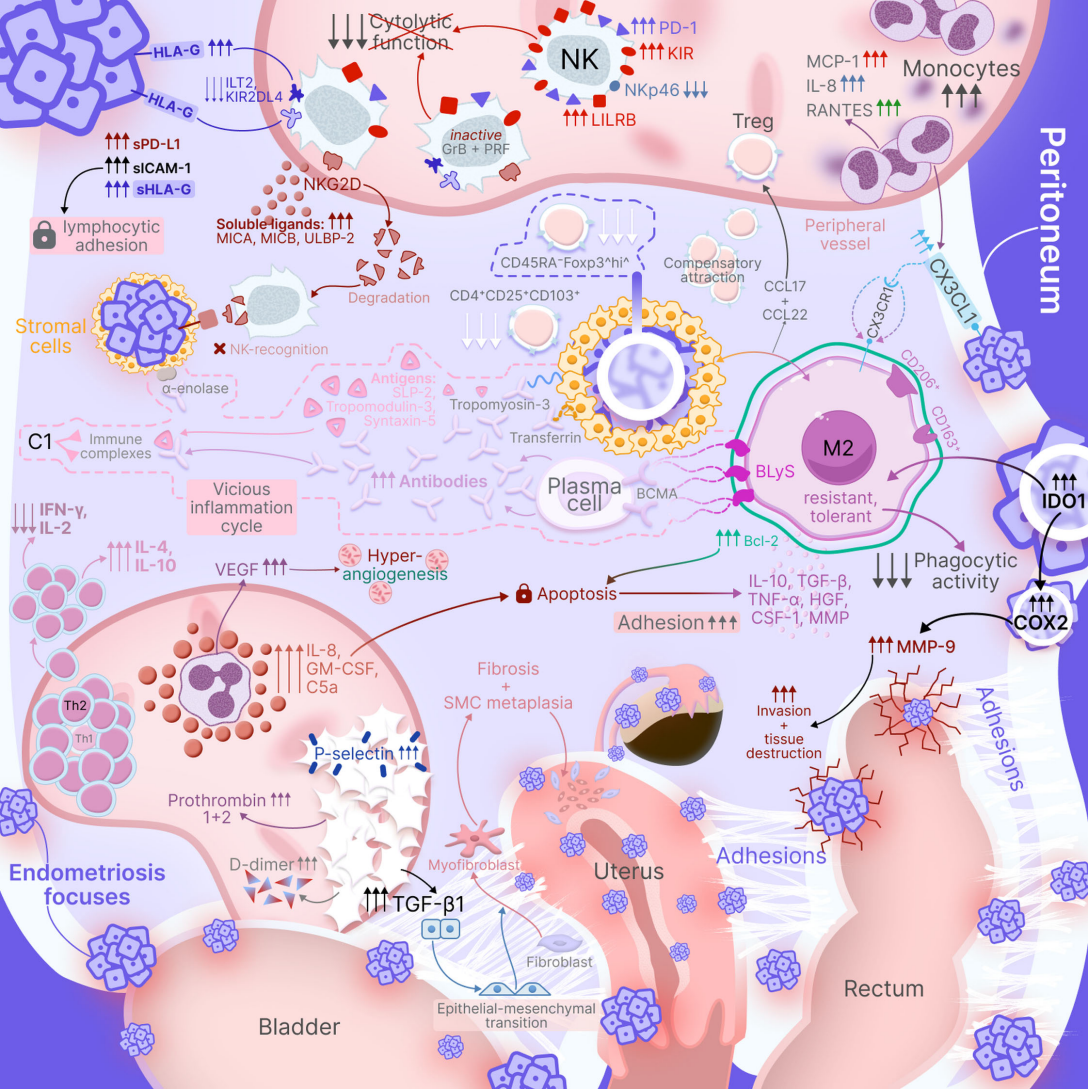
Immunopathogenesis of deep infiltrative endometriosis. The diagram depicts maximal immune dysregulation including: massive systemic monocyte activation with peak concentrations of chemokines (MCP-1, IL-8, RANTES, fractalkine) and M2 polarization; Bcl-2-expressing apoptosis-resistant M2 macrophages (CD163^+^/CD206^+^) as constant source of TNF-α, IL-10, growth factors (HGF, CSF-1), and MMPs; complete loss of neutrophil cytotoxic function with maximal VEGF-mediated hyperangiogenesis; NK cell exhaustion with maximum inhibitory receptors (KIR, LILRB) and PD-1 expression indicating exhaustion phenotype; paradoxical deficiency of active Tregs (CD45RA-Foxp3^hi^) in lesions with compensatory CCL17/CCL22-mediated recruitment; maximal Th2 shift systemically (IL-4/IL-10↑↑↑, IFN-γ/IL-2↓↓↓); complete autoimmune spectrum with BLyS-mediated plasma cell hyperplasia producing multiple autoantibodies and immune complex-complement activation; IDO1→COX-2→MMP-9 destructive axis driving maximal invasiveness; maximal immune evasion through HLA-G overexpression, soluble forms (sPD-L1, sHLA-G, sICAM-1), and total PD-L1 expression; soluble NKG2D ligands (MICA/MICB/ULBP-2) causing receptor degradation; and severe hypercoagulation state with platelet activation, TGF-β1 secretion, epithelial-mesenchymal transition, and extensive fibrosis with smooth muscle metaplasia.

Thus, rectocervical endometriosis pathogenesis represents a complex immunological process where the key role is played by combination of destructive changes (through IDO1/COX-2/MMP-9 signaling pathway) and paradoxical tolerogenic microenvironment formation, collectively ensuring both invasive lesion growth and their resistance to immune control (**Figure 5**).

### 7.4 Extragenital endometriosis

Extragenital endometriosis is a relatively rare phenomenon involving endometriotic lesions localized outside reproductive system organs. Most commonly affected are gastrointestinal tract (rectum and sigmoid colon, appendix) and urinary system (bladder, ureters). Generally, the closer an organ is located to the uterus, the higher the probability of involvement, explaining significant frequency of intestinal and urinary endometriosis (169).

Immunopathogenesis of extragenital forms is similar to that in deep infiltrative variants. The study by Scheerer et al. showed that immune infiltrates associated with extragenital endometriotic lesions lack NK cells (CD56^+^) (10). Their absence may be a key factor allowing endometriotic tissue to establish in atypical locations without immune destruction. Simultaneously, patients with extragenital endometriosis are characterized by elevated TGF-β levels in peritoneal fluid and blood serum (170). TGF-β, as noted, has strong immunosuppressive effect. It suppresses NK cell activity and other effector lymphocytes. Macrophages also demonstrate altered activation profile. M2 phenotype predominance maintains immune tolerance and tissue remodeling, creating favorable conditions for endometriotic cell persistence (37). Additionally, high PD-L1 molecule expression is found on macrophages and endometriotic cells in affected areas, additionally suppressing cytotoxic T-lymphocyte activity through PD-1 receptor interaction (171). Such changes enhance local immunosuppression and promote lesion evasion from immune surveillance. Angiogenesis role is also particularly important in extragenital forms context. VEGF expression is significantly elevated in lesions outside reproductive system, especially in intestinal and urinary localizations (172). Angiogenesis activity is closely associated with inflammatory cytokine levels such as IL-6 and TNF-α, forming a vicious cycle of chronic inflammation and disease progression.

Thus, extragenital endometriosis immunopathogenesis is characterized by profound innate and adaptive immunity disruptions. Key mechanisms include NK cell deficiency, macrophage dysfunction with immunosuppressive phenotype predominance, immune checkpoint (PD-L1) expression, VEGF-induced angiogenesis enhancement, and chronic inflammation. These processes jointly create favorable microenvironment for ectopic endometriotic tissue survival and progression in atypical localizations.

### 7.5 Comparative analysis and form-specific immunological signatures

All endometriosis forms share core immunopathogenetic mechanisms: macrophage accumulation with M2 polarization, reduced NK cytotoxicity, elevated pro-inflammatory cytokines (IL-1β, IL-6, TNF-α) alongside immunosuppressive mediators (IL-10, TGF-β). However, substantial form-specific differences exist.

Peritoneal endometriosis, being the most common form, demonstrates stage-dependent immune evolution: early stages (I-II) show M1 macrophage dominance with elevated IL-6/IL-8/TNF-α despite reduced cytotoxicity, while advanced stages (III-IV) exhibit M2 polarization driven by chronic DAMP stimulation and endometriotic cell-derived TGF-β/IL-10. NK cells retain pro-inflammatory cytokine production (TNF-α/IFN-γ) despite cytotoxic dysfunction—a paradoxical feature maintaining chronic inflammation. Ovarian endometriosis uniquely combines local immunosuppression (LGMNP1-driven M2 polarization, Treg expansion) with systemic inflammatory response and marked hemostasis activation, explaining both immune evasion and systemic complications. Mast cell-nerve fiber interactions contribute to neoinervation and pain syndrome formation. Adenomyosis demonstrates paradoxical Treg deficit with elevated Th17/Treg ratio and dominant HMGB-1/TLR4 activation (particularly in secretory phase), creating distinctly pro-inflammatory microenvironment. Fractalkine-mediated immune cell recruitment maintains inflammation chronicity. Deep infiltrative endometriosis is characterized by IDO1/COX-2/MMP-9 axis predominance driving tissue destruction and paradoxical Treg recruitment via CCL17/CCL22. NK cells exhibit PD-1^+^ exhaustion phenotype, suggesting therapeutic checkpoint inhibitor potential. Extragenital forms exhibit complete NK cell absence (CD56^+^) in lesion infiltrates and maximal TGF-β/PD-L1-mediated immunosuppression—unique features potentially enabling establishment in atypical anatomical sites.

These distinctions have therapeutic implications: peritoneal forms require stage-targeted approaches (anti-inflammatory in early stages, immunosuppression reversal in advanced); ovarian forms necessitate dual targeting of local and systemic inflammation; adenomyosis demands TLR4 pathway inhibition and Treg restoration; deep infiltrative forms may respond to IDO1/COX-2 blockade and checkpoint inhibitors; extragenital forms require NK cell restoration strategies.

## 8 Perspectives for targeted immunotherapy of endometriosis

With deepening understanding of endometriosis immunopathogenesis, promising experimental approaches for targeted immunotherapy are emerging. Unlike hormonal treatment methods that indirectly influence immune reactions, these new strategies aim at direct modulation of dysfunctional immune components causing endometriosis (40). Let us consider several advanced directions: immunomodulators, gene therapy, nanotechnology, cell-directed therapies (with emphasis on macrophages and NK cells), cytokine/chemokine inhibitors, and immune checkpoint targeting. Many of these approaches are at preclinical or early clinical stages, indicating a new era of precision immunotherapy for endometriosis.

### 8.1 Immunomodulating agents

Various immunomodulators capable of restoring immune balance in endometriosis have been studied. One approach is inflammation suppression using glucocorticoids. Given the side effects of steroids with long-term use, short-term glucocorticoid regimens were utilized, which improved fertility in endometriosis by suppressing pelvic inflammation (173). Another approach investigated is pentoxifylline, a phosphodiesterase inhibitor possessing immunomodulatory properties: it reduces TNF-α and IL-1 production by macrophages and enhances NK cell activity. Results of pentoxifylline application for pain reduction or fertility improvement are ambiguous; however, the study by Kamencic and Thiel showed that its addition to conservative surgery significantly improves pain syndrome control after 2–3 months observation compared to isolated surgical treatment (174). Reduction of endometriotic lesions in animal models has also been demonstrated with thalidomide therapy, known for its anti-TNF and anti-angiogenic effects, although its side effects limit clinical application (175). Given the increased presence of mast cells in endometriotic lesions, mast cell stabilizers such as sodium cromoglycate and leukotriene inhibitors (e.g., zafirlukast) are also being investigated as anti-inflammatory agents (78).

In preclinical studies, oral ketotifen administration significantly suppressed the development of hyperalgesia and reduced endometriotic cyst volume in rats (176). A promising therapeutic direction involves JAK3 inhibitors (JANEX-1) that specifically target mast cell activation pathways (177). Given the central role of MCs in coordinating inflammatory and neurogenic responses in endometriosis, as well as their involvement in the JAK/STAT pathway, these approaches may simultaneously address both lesion progression and pain generation (77).

### 8.2 Cytokines and chemokines as targeted therapy targets

Anti-TNF therapy is one of the most extensively studied due to TNF’s central role in inflammation development. In a pilot randomized controlled trial, infliximab did not show significant pain reduction in endometriosis compared to placebo, but subgroup analysis indicated that patients with initially elevated TNF levels demonstrated positive effects (178). Another approach is IL-6 blockade (for example, tocilizumab): IL-6 promotes ectopic tissue survival and suppresses NK cells, so its inhibition may restore immune attack on lesions. In animal experiments, tocilizumab significantly reduced endometriotic lesion volume and caused ectopic endometrial atrophy (179). IL-1 inhibitors (particularly soluble IL-1 receptor type II) have also shown effects on endometriotic lesions in animal models, likely through suppression of IL-1-induced angiogenesis and IL-6 production (180). Suppression of endometriotic implant growth in experiments using murine disease models has been observed with NK cell activation through IL-12 injections (181). Additionally, anti-IL-8 approaches may be valuable, considering IL-8’s role in neutrophil recruitment and angiogenesis (155). Blocking CCL2 (MCP-1) or its receptor CCR2 may prevent excessive monocyte recruitment to lesions; CCR2 antagonist showed reduced lesion establishment in mouse model (182). TGF-β1 deficiency in host suppressed endometriotic lesion development in murine endometriosis xenotransplantation model: median lesion mass was reduced 11-fold, and myofibroblast numbers decreased by 47% compared to control group (183).

### 8.3 Gene therapy, nanotechnology and nanomedicine

In preclinical trials, chitosan nanoparticles were used for pigment epithelium-derived factor (PEDF) gene delivery, which produced antiangiogenic protein capable of slowing endometriosis development (184). Later, Li Z et al. created poly (lactic-co-glycolic acid) (PLGA) nanoparticles loaded with anti-cytotoxic T-lymphocyte-associated protein 4 (CTLA-4) antibody (immune checkpoint inhibitor) (185). Another nanotechnological approach involved using nanovesicles derived from M1 macrophages (M1NVs): these are exosome-like particles collected from proinflammatory M1-polarized macrophages (186). Nanoparticles are also used for photothermal therapy: researchers loaded endometriotic lesions with heat-generating nanoparticles that, when exposed to near-infrared light, selectively destroy lesions with heat while preserving surrounding tissues (187). A different approach was chosen by Matsuzaki S et al., developing aquaporin-2 (AQP2) gene inhibition strategy, which participates in ectopic endometrial cell survival (188). Additionally, microRNA therapy is being studied: miR-200c, a tumor-suppressive microRNA capable of suppressing epithelial-mesenchymal transition, was delivered to endometriotic lesions by polymeric nanoparticles, leading to 1.5-fold reduction in lesion volume in rat model (189).

### 8.4 Macrophage and natural killer cell modulation

As described above, macrophages and NK cells play central roles in endometriosis immunopathology, so therapy directed at modulating their function comes to the forefront in experimental treatment approaches. Peritoneal macrophages in endometriosis are often “captured” by lesions for growth support—characterized by high scavenger receptor expression, growth factor secretion, and low phagocytic activity. One promising target is the CD47-SIRPα axis. Ectopic endometrial cells overexpress CD47, which binds to SIRPα on macrophages, suppressing phagocytosis (190). By blocking CD47, macrophage ability to engulf and destroy endometriotic cells can be restored. Another macrophage-oriented approach is exosome blockade. Lesion cells release exosomes that can induce macrophages to polarize toward M2 phenotype and reduce their phagocytic capacity (191). *In situ* macrophage reprogramming is another potential strategy. Complement system modulation can influence macrophages: blocking C1q (complement protein elevated in peritoneal fluid in endometriosis) showed prevention of C1q-induced M2 polarization (142). NK cells express various inhibitory receptors (KIRs, leukocyte immunoglobulin-like receptor subfamily B [LILRB], PD-1, etc.) that are elevated in endometriosis (74, 192, 193). Blocking these checkpoints may restore NK cell activity. PD-1/PD-L1 blockade appears particularly promising. Endometriotic lesions have been shown to express PD-L1, which can bind to PD-1 on NK and T cells, suppressing their effector properties. Preclinical studies suggested that anti-PD-1 therapy may reduce lesion size and improve immune cell infiltration in endometriosis models (194). In endometriosis immunotherapy context, probiotics also play significant roles, influencing NK cells: oral Lactobacillus gasseri showed NK cell activation and endometriotic lesion size reduction in mouse model (195). This phenomenon likely reflects common pathogenetic mechanisms underlying observed relationship between intestinal dysbiosis and gynecological diseases, particularly endometriosis.

### 8.5 Inhibition of the complement system

Dysregulation of complement plays a key role in the pathogenesis of endometriosis: reduced levels of the embryotrophic factor iC3b may explain associated infertility, whereas elevated SC5b-9 promotes survival of ectopic cells (13). Studies in C3-deficient mice confirmed that C3 absence significantly reduces endometriotic cyst formation, and C3a maintains inflammation through mast cell activation (80, 144).

C3 inhibitors (AMY-101) appear to be the most promising approach, interrupting the inflammatory cascade and potentially restoring NK cell cytotoxicity (142). C5 blockade (eculizumab) is safe but does not address C3-dependent mechanisms (142). An additional target is the C1q–gC1qR interaction, which mediates lesion vascularization independent of complement activation (146). This direction requires clinical trials to determine optimal points of therapeutic intervention.

### 8.6 Th17/Treg pathway targeting

Endometriosis is often described as immune tolerance state (high Treg activity) paradoxically coexisting with inflammation (Th17 and others). Therapies for correcting this Treg/Th17 imbalance are being developed. One innovative concept is Treg cell reprogramming. By inhibiting Treg-associated checkpoints (CTLA-4, as noted with PLGA nanoparticles, or PD-1 on Treg), lesion size can be reduced (185). Since retinoic acid-related orphan receptor-γ (RORγt) is the main regulator of Th17 differentiation, RORγt inhibitors (in development for autoimmune diseases) could potentially reduce Th17 cell generation in endometriosis and thus decrease IL-17-mediated pathology (196). Tolerogenic dendritic cell-based vaccines are considered a promising approach aimed at changing Treg/Th17 balance toward Treg. In this case, dendritic cell generation protocol is based on their loading with autologous endometrial antigens. Such tolerogenic dendritic cells would subsequently induce antigen-specific Tregs that could migrate to lesions and mitigate inflammation (197).

Potential novel targets include galectins (proteins regulating immune cell apoptosis and serving as ligands for inhibitory checkpoint receptors), fractalkine (CX3CL1) and its receptor, and vitamin D receptor pathways. Researchers suggest vitamin D’s ability to reduce pain and inflammation in endometriosis (198). Thus, endometriosis immunotherapy represents actively developing direction. Existing hormonal and surgical treatment methods do not address underlying immune dysfunction, while new methods are aimed precisely at correcting immunological disease mechanisms. It is expected that within the next decade, some of these experimental approaches will be implemented in clinical practice, especially for patients with resistant disease forms or contraindications to hormonal therapy.

## 9 Discussion

In contemporary medicine, endometriosis is viewed not simply as endocrine or gynecological disorder, but as complex disease fundamentally based on intricate interactions between innate and adaptive immune systems and ectopic endometrial tissue. The conducted analysis convincingly demonstrates that endometriosis pathogenesis is determined by disruptions in both innate immunity (macrophages, neutrophils, NK cells, complement) and adaptive immunity (T- and B-lymphocytes, especially Th17 and Treg subpopulations). Involvement of these two immune system components is traced through all disease development stages: innate immune cells form inflammatory microenvironment ensuring survival of implanted endometrial cells, while adaptive immunity dysfunction, manifesting as reduced T-cell cytotoxic activity and immunological tolerance formation via Treg, allows these cells to avoid elimination. These processes jointly contribute to endometriotic lesion implantation, growth, angiogenesis, and progressive tissue damage, creating a closed cycle of inflammation and hormonal stimulation.

A common feature of all endometriosis forms and stages is immune system suppression by ectopic tissue—the immune escape phenomenon, which brings this disease closer to tumor processes and explains why endometriosis is often characterized as having “malignant features” while being benign. All endometriosis forms share certain immunopathogenetic mechanisms: peritoneal macrophage accumulation and their polarization toward regenerative phenotype, reduced NK cell cytotoxicity, elevated local proinflammatory cytokine levels (IL-1β, IL-6, IL-8, TNF-α) with simultaneous increases in immunosuppressive mediators (IL-10, TGF-β), collectively creating favorable conditions for ectopic tissue development. These common pathogenetic mechanisms determine clinical disease manifestations such as chronic inflammation and pain syndrome.

Nevertheless, specific immunological features of different endometriosis forms must be noted. Deep infiltrative endometriosis is characterized by more pronounced cell-mediated inflammatory response with high IL-12, IFN-γ, and chemokine activity. Ovarian endometriomas are accompanied by systemic inflammation development and oxidative stress in cyst content. Peritoneal (superficial) lesions may manifest less pronounced immune changes but depend more on local peritoneal immunity failure. Understanding these differences has important significance for both individualized therapeutic approach development and explaining clinical manifestation diversity. Immune profile may substantially differ in patients with isolated ovarian cyst versus fibrous deep infiltrative endometriotic lesions, despite both conditions being classified as “endometriosis”.

It should be particularly emphasized that correlation of immunological changes with disease stage and symptom severity opens perspectives for using immune markers as biomarkers for endometriosis diagnosis and prognosis. Integration of immunological data (cytokine profiles, immune cell phenotypes) with clinical staging may improve disease course prediction accuracy and optimize treatment strategy selection. Thus, a patient with initial disease manifestations but pronounced inflammatory immune profile may require more careful monitoring or additional immunotropic therapy to prevent process progression. Meanwhile, patients with advanced endometriosis, but less pronounced immune dysregulation, may exhibit favorable response to conservative therapy.

Contemporary scientific data indicate that endometriosis lies at the intersection of endocrinology and immunology—estrogen dependence and immune dysfunction are interconnected in disease pathogenesis. From research perspective, priority directions include further study of molecular interactions between ectopic endometrial cells and immune cells, particularly mechanisms by which endometriotic lesions reprogram macrophages or induce Treg expansion. Equally important is searching for reliable non-invasive disease biomarkers. Considering that many immune mediators, including cytokines and immune cell microRNAs, are detectable in blood, developing diagnostic panels of such markers may fundamentally change approaches to endometriosis diagnosis (currently predominantly surgical) and therapy efficacy monitoring.

Additional research is needed to evaluate long-term effects of immunomodulating therapy in endometriosis patients, especially in the context of immunotherapeutic approach development. Safety profile, fertility impact, and recurrence rates should become key criteria for evaluating new treatment method efficacy.

From practical standpoint, deepened understanding of endometriosis immunopathogenesis opens new perspectives in treating this disease. Clinicians should consider that endometriosis patients often have systemic inflammation, justifying need for comprehensive approach to managing such patients, including correction of concomitant inflammatory diseases, diet optimization, and stress level reduction, which significantly influence immune system. Combined treatment methods appear justified, particularly supplementing hormonal therapy with immunomodulating drugs in individual clinical cases (for example, pentoxifylline for fertility improvement or, prospectively, biological drugs—cytokine inhibitors for more effective pain syndrome control). Individualizing treatment approaches depending on disease phenotype is advisable: “immunological profiling” methodology may become component of personalized therapy, allowing prediction of both experimental immunotherapeutic method efficacy and standard treatment regimen effectiveness.

Conceptually, endometriosis can be viewed as disease fundamentally based on inadequate immune response to menstrual trauma. Endometrial fragments maintain viability in ectopic localizations not only due to retrograde menstruation and estrogenic stimulation, but also as result of immune system failure, which not only fails to eliminate ectopic cells but, paradoxically, supports their existence. This process involves both innate immunity (through inflammation and tissue remodeling) and adaptive immunity (through insufficient immune surveillance and tolerance formation). Integration of these scientific principles opens paths to developing new diagnostic and therapeutic approaches considering both hormonal and immunological pathogenesis aspects of endometriosis.

Promising treatment methods successfully integrating immunomodulation (as supplement or alternative to hormonal therapy) open possibilities for more effective disease control, reduced recurrence rates, and reproductive function preservation. In the coming decade, implementation of many discussed experimental strategies in clinical trials is expected, which should bring us closer to an era when immunotherapy becomes integral component of standard endometriosis treatment complex. Ultimately, bridging the gap between fundamental science and clinical practice in endometriosis immunology will improve quality of life for millions of women suffering from this disease and possibly transform endometriosis from chronic recurrent condition to controllable or even curable disease.

### 9.1 Limitations

This review has several limitations that warrant consideration. First, the narrative synthesis approach precluded quantitative meta-analysis of effect sizes and prevented statistical assessment of heterogeneity across studies. Second, substantial methodological heterogeneity existed among included studies regarding immune parameter assessment techniques (flow cytometry, ELISA, PCR, immunohistochemistry), disease classification systems, patient selection criteria, and control group definitions, complicating direct comparisons and potentially contributing to result contradictions. Third, the predominance of retrospective and cross-sectional study designs among included research limits causal inference regarding immune dysregulation and disease progression. Fourth, the search was limited to four databases (PubMed, Scopus, Web of Science, Google Scholar), potentially missing relevant publications in other databases or grey literature. Fifth, this review was not prospectively registered, and no protocol was published prior to initiation. Sixth, potential publication bias favoring positive findings and statistically significant results may exist, as studies with negative or null results are less likely to be published. Finally, most research focused on peritoneal and ovarian endometriosis, with limited data on extragenital forms and early-stage disease, restricting generalizability of findings across all endometriosis phenotypes.

Despite these limitations, this comprehensive synthesis of 198 high-quality publications provides valuable insights into endometriosis immunopathogenesis, identifies critical knowledge gaps, and offers evidence-based directions for future research and therapeutic development.
