# Supplementary figures and images for: Endometriosis as an immune-mediated disease: pathogenetic mechanisms and therapeutic strategies
Source: Front Immunol. 2025 Dec 18;16:1727183. doi: 10.3389/fimmu.2025.1727183 (PMC12756115; doi:10.3389/fimmu.2025.1727183)

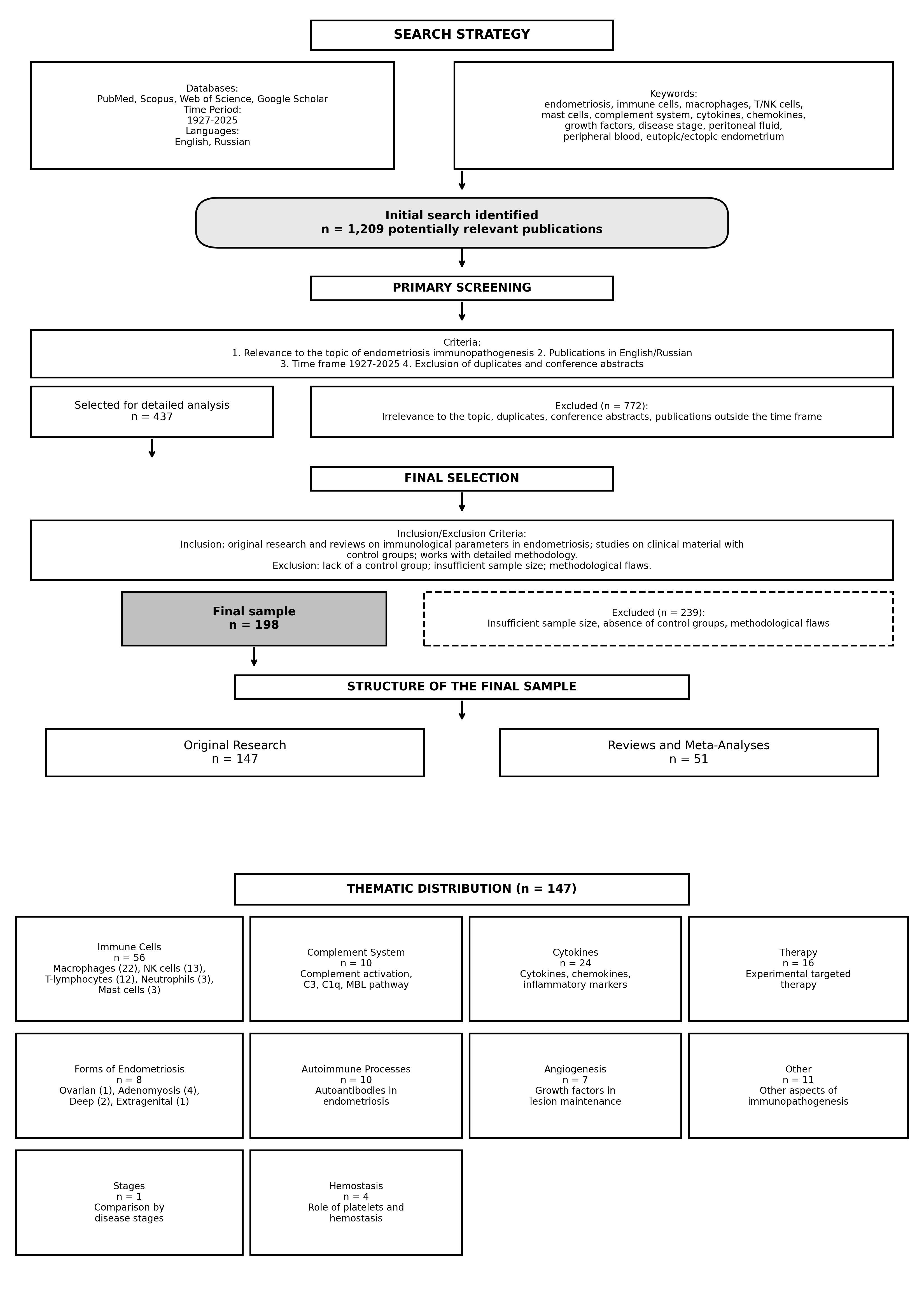

Supplement: Supplementary file 1 [file Image1.png]
